# Cancer cell-intrinsic expression of MHC II in lung cancer cell lines is actively restricted by MEK/ERK signaling and epigenetic mechanisms

**DOI:** 10.1136/jitc-2019-000441

**Published:** 2020-04-19

**Authors:** Alexander J Neuwelt, Abigail K Kimball, Amber M Johnson, Benjamin W Arnold, Bonnie L Bullock, Rachael E Kaspar, Emily K Kleczko, Jeff W Kwak, Meng-Han Wu, Lynn E Heasley, Robert C Doebele, Howard Y Li, Raphael A Nemenoff, Eric T Clambey

**Affiliations:** 1 Medical Oncology, Hunter Holmes McGuire VA Medical Center, Richmond, Virginia, USA; 2 Anesthesiology, University of Colorado Denver - Anschutz Medical Campus, Aurora, Colorado, USA; 3 Medicine, University of Colorado Denver - Anschutz Medical Campus, Aurora, Colorado, USA; 4 Craniofacial Biology, University of Colorado Denver - Anschutz Medical Campus, Aurora, Colorado, USA; 5 VA Eastern Colorado Health Care System, Denver, Colorado, USA; 6 Internal Medicine, Division of Pulmonary Disease and Critical Care Medicine, Virginia Commonwealth University, Richmond, Virginia, USA; 7 Medical Service, Pulmonary Section, Hunter Holmes McGuire VA Medical Center, Richmond, Virginia, USA

**Keywords:** immunology, interferon, tumours

## Abstract

**Background:**

Programmed death 1/programmed death ligand 1 (PD-1/PD-L1) targeted immunotherapy affords clinical benefit in ~20% of unselected patients with lung cancer. The factor(s) that determine whether a tumor responds or fails to respond to immunotherapy remains an active area of investigation. We have previously defined divergent responsiveness of two KRAS-mutant cell lines to PD-1/PD-L1 blockade using an orthotopic, immunocompetent mouse model. Responsiveness to PD-1/PD-L1 checkpoint blockade correlates with an interferon gamma (IFNγ)-inducible gene signature and major histocompatibility complex class II (MHC II) expression by cancer cells. In the current study, we aim to identify therapeutic targets that can be manipulated in order to enhance cancer-cell-specific MHC II expression.

**Methods:**

Responsiveness to IFNγ and induction of MHC II expression was assessed after various treatment conditions in mouse and human non-small cell lung cancer (NSCLC) cell lines using mass cytometric and flow cytometric analysis.

**Results:**

Single-cell analysis using mass and flow cytometry demonstrated that IFNγ consistently induced PD-L1 and MHC class I (MHC I) across multiple murine and human NSCLC cell lines. In contrast, MHC II showed highly variable induction following IFNγ treatment both between lines and within lines. In mouse models of NSCLC, MHC II induction was inversely correlated with basal levels of phosphorylated extracellular signal-regulated kinase (ERK) 1/2, suggesting potential mitogen-activated protein (MAP) kinase-dependent antagonism of MHC II expression. To test this, cell lines were subjected to varying levels of stimulation with IFNγ, and assessed for MHC II expression in the presence or absence of mitogen-activated protein kinase kinase (MEK) inhibitors. IFNγ treatment in the presence of MEK inhibitors significantly enhanced MHC II induction across multiple lung cancer lines, with minimal impact on expression of either PD-L1 or MHC I. Inhibition of histone deacetylases (HDACs) also enhanced MHC II expression to a more modest extent. Combined MEK and HDAC inhibition led to greater MHC II expression than either treatment alone.

**Conclusions:**

These studies emphasize the active inhibitory role that epigenetic and ERK signaling cascades have in restricting cancer cell-intrinsic MHC II expression in NSCLC, and suggest that combinatorial blockade of these pathways may engender new responsiveness to checkpoint therapies.

## Background

In the USA, lung cancer has an incidence of 225 000 patients every year leading to approximately 160 000 deaths.^1^ Over the last several years, the development of targeted therapeutics for the treatment of patients with non-small-cell lung cancer (NSCLC) with specific genetic changes, such as epidermal growth factor receptor (EGFR) mutations, echinoderm microtubule-associated protein like-4-anaplastic lymphoma kinase (EML4/ALK) fusion and c-ros oncogene 1 (ROS1) fusions, have led to exciting new treatment options for these patients. Unfortunately, virtually all lung cancers with driver mutations ultimately develop resistance to targeted therapies.^2^ Another recent development in the treatment of NSCLC involves the use of antibodies targeting immune checkpoint molecules including PD-1 or its ligand PD-L1, which can lead to durable responses in around 15%–20% of unselected patients with advanced NSCLC.^3 4^ Despite promising results with these immunotherapy-based therapies, the majority of patients with lung cancer fail to respond to this intervention. Extensive ongoing research efforts continue to define predictors of response to checkpoint therapy, with tumor mutational burden and characteristics of the tumor microenvironment (TME), including lymphocytic infiltration and an interferon gamma (IFNγ) responsive gene signature (ie, PD-L1 expression and the induction of antigen presentation machinery, MHC class I and class II molecules) positively correlated with response to therapy.^5–9^ Additional studies have further identified mechanisms of resistance to checkpoint therapy, including mutations in genes associated with IFNγ signaling [janus kinase (JAK)1 and JAK2] and antigen presentation (beta-2-microglobulin).^10^

Our group has previously analyzed determinants of response to immune checkpoint blockade, using orthotopic implantation of KRAS mutant NSCLC lines into syngeneic hosts.^11^ These studies have demonstrated that the CMT167 cell line is both sensitive to checkpoint blockade with PD-1/PD-L1 antibodies and exhibits an IFNγ responsive phenotype in vivo. Conversely, the Lewis lung carcinoma (LLC) cell line is resistant to checkpoint blockade and has a blunted IFNγ-inducible gene signature in vivo. Notably, while both CMT167 and LLC cells show induction of some IFNγ-inducible genes in vivo, including PD-L1, CMT167 cells showed a unique induction of genes encoding the MHC class II antigen presentation and processing pathway.^12^

In this study, we aimed to characterize the determinants of divergent IFNγ responsiveness between these tumor lines in vitro in order to gain mechanistic insights that may lead to novel approaches to enhance IFNγ sensitivity and thus make more tumors susceptible to PD-1/PD-L1 checkpoint blockade, with an emphasis on MHC II regulation. Our studies point to a critical requirement for epigenetic and post-translational-dependent mechanisms that actively restrict IFNγ induction of the MHC class II machinery in NSCLC.

## Methods

### Cell lines

Murine cell lines: CMT167 and LLC cells stably expressing firefly luciferase were used in this study.^11^ Luciferase-expressing LLC cells were purchased from Caliper Life Sciences (LL/2-luc-M38). CMT167 cells were originally obtained from the lab of Alvin Malkinson (University of Colorado, Denver). The CMT167 cells contained a firefly luciferase that was stably transfected into the cells as previously described.^13^ CMT167 and LLC cells were maintained in DMEM with 4.5 g/L glucose containing 10% fetal bovine serum (FBS), 100 U/mL penicillin and 100 µg/mL streptomycin, and were cultured at 37°C at 5% CO_2_. Cells were regularly analyzed for morphology and functional characteristics to assure that the lines maintained consistent phenotypic characteristics for the duration of the study. To avoid cross-contamination, all cell lines used were not maintained in long-term cultures but rather maintained as frozen stocks and only kept in live culture for 2–4 weeks period. The EA1 cell line was derived from C57BL/6 mice treated with adenovirus containing Cas9 and guide RNAs leading to generation of tumors containing an EML4-ALK fusion as previously described.^14^ A second EML4-ALK cell line (EA2) was derived from a p53 deficient mouse treated with a Crispr/Cas9 adenovirus construct engineered to create an EML4-ALK translocation.^15^ EA2 cells were kindly provided by Andrea Ventura, Memorial Sloan Kettering Cancer Center. Both EML4-ALK fusion cell lines (EA1 and EA2) were confirmed to contain EML4-ALK fusion at the genomic level, demonstrated sensitivity to the ALK inhibitor alectinib and formed viable tumors when directly injected to the lungs of C57BL/6 mice. EA1 and EA2 cell lines were maintained in Roswell Park Memorial Institute (RPMI) medium containing 10% FBS, 100 U/mL penicillin and 100 µg/mL streptomycin, and were cultured at 37°C at 5% CO_2_. We followed similar procedures for maintaining the integrity of our cell lines as our group has previously reported.^14 16^

Human cell lines. Human NSCLC cell lines included: H460 (KRAS^Q61K^, large cell lung cancer^17^), H1299 (NRAS^Q61K^ mutant^18^), H1650 (EGFR^E746_A750del^ 19), HCC827 (EGFR^E746_A750del^ 20) and HCC78 (SCL34A2-ROS1 fusion^21^), all obtained from the lab of Robert Doebele, University of Colorado; Doebele’s lab obtained these cell lines as previously published.^22^ Ponatinib-resistant 1 (PR1) is a coiled-coil domain containing 6 (CCDC6)-rearranged during transfection (RET) fusion-positive cell line that was originally derived as a ponatinib-resistant line using dose escalation strategies from its parental line LC2-AD as published by Doebele’s lab.^23 24^ H1703 is a platelet-derived growth factor receptor alpha/fibroblast growth factor receptor 1 (PDGFRα/FGFR1)-amplified cell line obtained from the Doebele lab, and their prior work and characterization of this cell line has been published.^24–26^ Additional human lung adenocarcinoma cell lines, obtained from Lynn Heasley, included: H358 cells (KRAS^G12C^ mutation), A549 cells (KRAS^G12S^ mutation) and SW1573 cells (KRAS^G12C^ mutation). Cells were periodically tested for mycoplasma infection, maintained as frozen stocks and cultured for only 2–4 weeks before use in experiments. Human lung adenocarcinoma cell lines were maintained in RPMI 1640 (#10–040-CV, Corning) containing 10% FBS at 37°C in a humidified 5% CO_2_ atmosphere.

### Flow cytometric analysis

CMT167, LLC cells, EA1 and EA2 cells were subjected to various treatments. After described treatment period, cells were trypsinized, collected and washed. For surface stains, cells were resuspended in flow cytometry staining buffer^27^ and stained for 30 min with indicated antibodies. For intracellular stains, collected cells were resuspended in BD Biosciences Lyse/Fix buffer (catalog 558049) for 30 min at 37°C. For studies comparing intracellular signaling, samples were subjected to fluorescent barcoding.^28^ Barcoding was performed by staining fixed cells with varying concentrations of Ghost 510 and Ghost 450 viability dyes from Tonbo Biosciences (catalog (cat) # 13–0863 T100 and 13–0870 T100). After barcoding, all samples were combined and permeabilized for 20 min on ice using BD Biosciences perm buffer III (cat # 558050). Cells were then washed and stained with intracellular antibodies for 30 min. Cells were analyzed using a BioRad ZE5 flow cytometer, using beads as compensation controls (OneComp eBeads, Invitrogen/ThermoFisher). Data were analyzed using Kaluza software. Specific antibodies and reagents for flow cytometric analysis included: anti-pERK 1/2 (T202/Y204) PE-eFluor 610 Clone MILAN8R (eBioscience/ThermoFisher, cat # 61-9109-41), anti-STAT1 PE Clone 1/Stat1 (BD Biosciences cat # 558537), ghost dye violet 450 (Tonbo Biosciences, cat # 13–0863 T100), anti-mouse PD-L1 PE-Cy7 clone MIH5 (eBioscience/ThermoFisher, cat # 25-5982-82), anti-mouse MHC II PE clone M5/114.15.2 (eBioscience/ThermoFisher, cat # 12-5321-82), and anti-mouse MHC I allophycocyanin (APC) clone 34-1-2S (eBioscience/ThermoFisher, cat # 17-5998-80). Antibodies for the analysis of human cell lines included: anti-Human Leukocyte Antigen – DR (HLA-DR) APC clone L243 (BioLegend cat # 307609), anti-HLA-DQ1 PE clone HLA-DQ1 (BioLegend cat # 318105), anti-HLA A, B, C APC-Fire 750 clone W6/32 (BioLegend cat # 311443) and anti-PD-L1 PE-Cy7 clone MIH1 (eBioscience/ThermoFisher, cat # 25-5983-41). Analysis of HLA-DR expression in A549 and H358 cells was done prestaining cells with human FcR Binding Inhibitor (eBioscience 14-9161-71) for 15 min, followed by staining with LIVE/DEAD Fixable Aqua Stain (Invitrogen L34966) and anti-HLA-DR APC. These samples were subjected to postacquisition compensation using VersaComp Antibody Capture Bead Kit (Beckman Coulter #B22804) and analyzed using Kaluza Software (Beckman Coulter).

### Mass cytometry staining and processing

CMT167 and LLC cells were cultured for 48 hours under basal conditions or with 100 ng/mL IFNγ. Cells were then collected with trypsin, washed with PBS and resuspended in cisplatin (Fluidigm). Cells were incubated in cisplatin for 5 min, with the staining reaction quenched with MaxPar cell staining buffer (Fluidigm). Cells were subjected to barcoding using the Fluidigm Cell-ID 20 Plex Palladium barcoding kit according to manufacturer’s protocol (Fluidigm cat #201060). After barcoding, all samples were combined, and stained with a cocktail of metal-conjugated and fluorescently conjugated antibodies to detect cell surface proteins for 30 min at room temperature (RT) (all metal-conjugated antibodies from Fluidigm). Cells were then washed and incubated with secondary antibodies (anti-FITC, anti-PE, anti-APC, anti-Biotin) for 30 min at RT. Cells were permeablized with FoxP3 Fix/Perm buffer overnight at 4°C. Cells were then washed and incubated with antibodies to detect intracellular antigens for 2 hours at 4°C. Cells were washed, resuspended in intercalator solution, washed and resuspended with equilibration beads prior to collection on a Helios mass cytometer (Fluidigm).^29^

### Mass cytometry data analysis

Samples were normalized using NormalizerR2013b_MacOSX and debarcoded using SingleCellDebarcoderR2013b_MacOSX, with both programmes downloaded from the Nolan laboratory GitHub page (https://github.com/nolanlab) (as in^29^). Normalized, debarcoded data were then subjected to traditional Boolean gating in FlowJo 10.4.2, identifying all viable nucleated (^191^Iridium (Ir)+, ^193^Ir+, ^195^Platinum (Pt)-) events. All viable nucleated events were imported into the Cytofkit analysis pipeline.^30^ Data were subjected to clustering: (1) with 27 markers selected for clustering (online supplementary table 1), (2) use of the merge method ‘ceil’ (10 000 events per sample), (3) files transformed via the cytofAsinh method, and (4) clustered with the PhenoGraph algorithm, using t-Distributed Stochastic Neighbor Embedding (tSNE) as the visualization method.^30^ Results were visualized via the R package ‘Shiny’ where labels, dot size and cluster color were customized according to cluster identity or the expression of various cellular markers. Expression of individual parameters, colored according to relative expression intensity were generated with the Cytofkit ‘Shiny’ interface.

10.1136/jitc-2019-000441.supp1 Supplementary data

### Treatments

Recombinant mouse IFNγ (Peprotech, #315–05) and human IFNγ (eBioscience, # 14-8319-80 or Peprotech # 300–02) were used respectively to stimulate murine and human cell lines. Cells were further treated with various inhibitors including IκB kinase (IKK)-16 (Cayman Chemicals, #13313), ruxolitinib (LC laboratories, #R-6688), cobimetinib (Cayman Chemicals, #19563), trametinib (Selleckchem, #S2673) and trichostatin A (TSA) (Sigma-Aldrich, # T8552). Compounds were diluted in a manner consistent with manufacturer recommendations.

### Quantitative real-time-PCR

For quantitative real-time-PCR analysis, treated cells were harvested and homogenized in 350 µL of Buffer RLT (RNeasy, Qiagen). Total RNA was isolated using an RNAeasy Kit (Qiagen) and reverse transcription was performed on 1 µg of total RNA using qScript cDNA SuperMix (Quanta BioSciences 95 048–025). Real-time PCR analysis was conducted in triplicate in a C1000 Touch Thermal Cycler (Bio-Rad) using Power SYBR Green PCR Master Mix (Applied Biosystems 4367659). The relative message levels of each target gene were normalized to GAPDH.^31^ Primers for genes as follows: Human GAPDH, Fwd: GTCAACGGATTTGGTCGTATTG, Rev: TGGAAGATGGTGATGGGATTT. Human CIITA, Fwd: CCTGGAGCTTCTTAACAGCGA, Rev: TGTGTCGGGTTCTGAGTAGAG.

### Software and statistical analysis

Flow cytometry data were analyzed using Kaluza software V.2.1.1 (Beckman Coulter) and GraphPad Prism software V.8. Mass cytometry data were analyzed using FlowJo V.10.2.4, R studio (V.1.0.136), the Cytofkit package (https://github.com/JinmiaoChenLab/cytofkit; Release 3.6), Excel (V.15.13), Adobe Illustrator.^20^ All statistical analyzes were done using GraphPad Prism software. Specific statistical tests performed are described in figure legends, with statistical significance defined if p<0.05.

## Results

**Figure 1:**
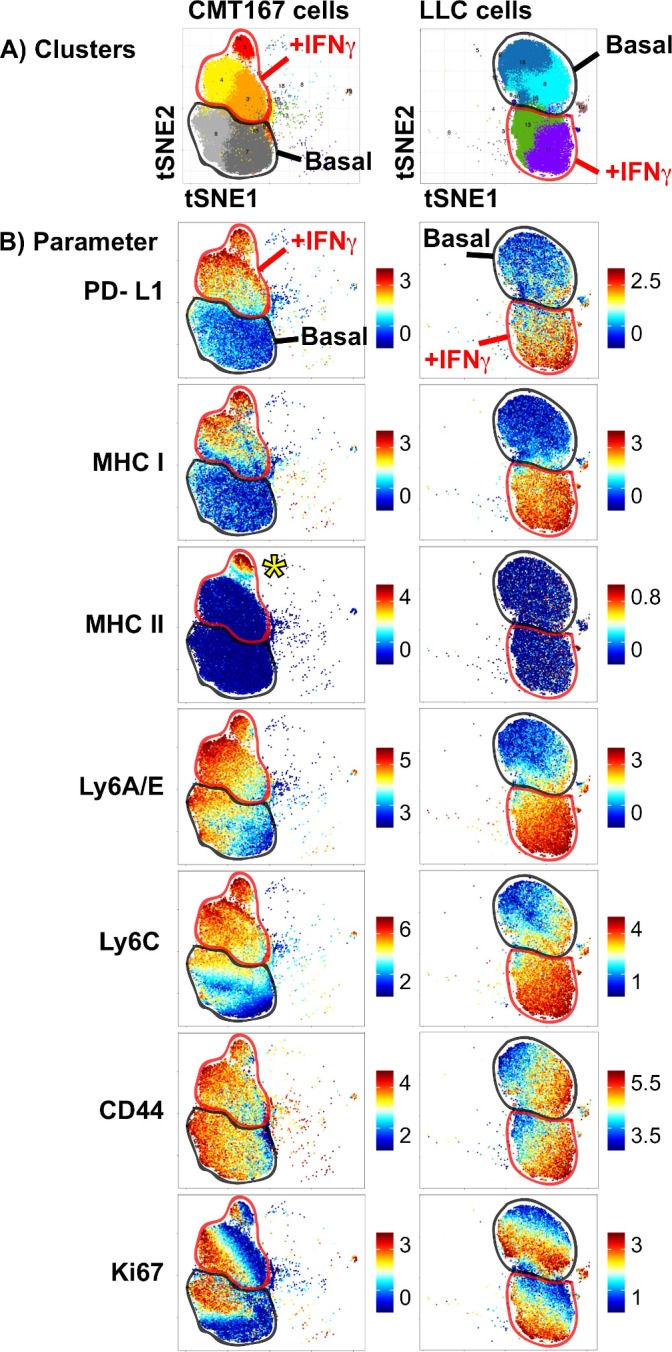
High-dimensional mass cytometry analysis of murine NSCLC cells stimulated with IFNγ. CMT167 and LLC cells were cultured under either basal or IFNγ stimulated conditions (100 ng/mL) for 48 hours, harvested and stained with a panel of metal-conjugated antibodies. (A) PhenoGraph analysis of CMT167 (left) and LLC cells (right), in which dominant cell clusters present under basal or IFNγ stimulation were identified. Cellular events were plotted on a tSNE plot, where each unique cluster is given a distinct color. (B) Analysis of protein expression in CMT167 (left) and LLC cells (right), with each plot depicting expression in cells cultured under basal (“basal”, outlined in thick black line on each plot) or IFNγ stimulated conditions (‘+IFNγ’, outlined in thick red line on each plot). Each row depicts expression of a different protein, with scale on the right side depicting range from minimum (bottom) to maximum (top), calculated based on the equation arcsinh (x/f), where x is the signal intensity and f=5. Yellow asterisk in MHC II plot for CMT167 cells identifies a subpopulation of IFNγ-treated cells that induce MHC II expression. PhenoGraph analysis was done on files that were normalized to equilibration beads, with all events defined as viable (^195^Pt-), nucleated (^191^Ir+ ^193^Ir+) events. CMT167 and LLC cells, under basal and IFNγ stimulated conditions were clustered together, using 10 000 events per sample and 27 markers. Data are from barcoded samples, with n=2 samples per condition. IFNγ, interferon gamma; LLC, Lewis lung carcinoma; NSCLC, non-small-cell lung cancer.

We previously reported that the CMT167 and LLC mouse models of NSCLC differ in their response to PD-1-targeted immunotherapy, with divergent IFNγ signatures in vivo.^31^ To further examine this, we assessed the ability of these cells to respond to in vitro IFNγ stimulation, measuring protein expression at the single-cell level. CMT167 and LLC cells were cultured under either basal or IFNγ stimulated conditions and subjected to mass cytometry, with cellular phenotypes characterized using the PhenoGraph clustering algorithm, visualized using the tSNE dimensionality reduction approach.^30^ In both CMT167 and LLC cell lines, IFNγ stimulation induced a shift in phenotypic clusters (top row, figure 1). In both CMT167 and LLC cells, IFNγ treatment resulted in the induction of PD-L1, MHC I, LyA/E and Ly6C proteins, with no impact on CD44 expression (figure 1). Despite the robust induction of IFNγ stimulated genes in both cell lines, CMT167 cells uniquely induced a subpopulation of cells that expressed MHC II (indicated by the yellow asterisk, in figure 1B ‘CMT167 cells, MHC II’). MHC II induction did not correspond to cell cycle status, defined by Ki67 expression (figure 1). These data indicate that MHC II induction uses distinct mechanisms from other IFNγ inducible targets. The partial induction of MHC II in CMT167 cells further suggests that additional phenotypic variation at the single-cell level may influence the kinetics or magnitude of MHC II induction.

**Figure 2:**
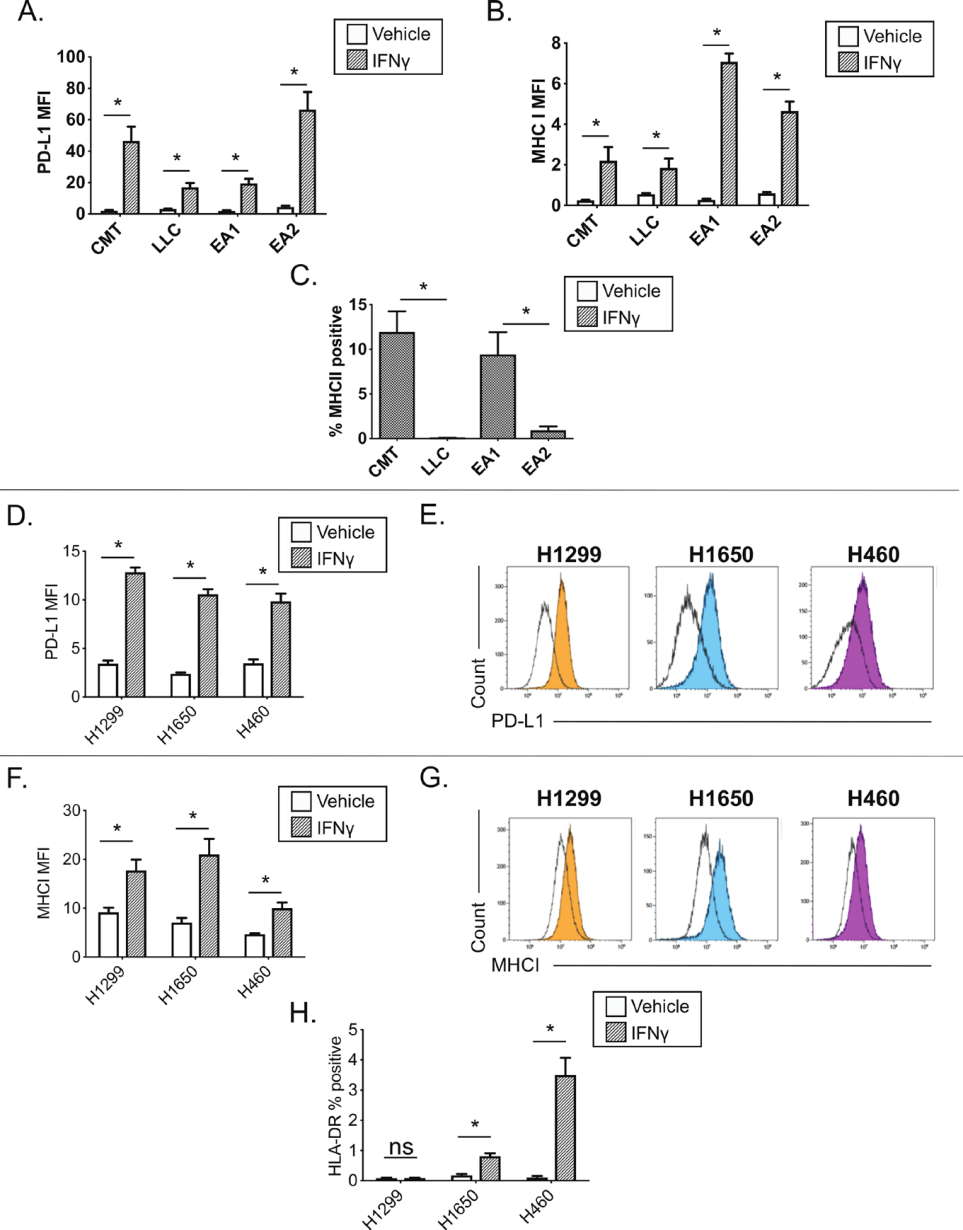
Interferon gamma responsiveness in mouse and human NSCLC cell lines. In A–C, cultured cell lines (CMT167, LLC, EA1, EA2) were treated with vehicle or 100 ng/mL IFNγ for 48 hours, and analyzed by flow cytometry for cell surface expression of (A) PD-L1, (B) MHC I, or (C) MHC II. Data for PD-L1 and MHC I are defined by median fluorescent intensity (MFI) of all cells, given the uniform induction of these proteins (online supplementary figure 1). MHC II expression is defined as the frequency of positive cells, based on MHC II induction in a subset of cells (online supplementary figure 2). Statistical analysis in (A, B) defined between vehicle and IFNγ treated samples using an unpaired T-test (statistical significance identified by *p<0.05). Statistical analysis in (C) compared the frequency of MHC II+events between cell lines with matched oncogene status (ie, KRAS mutant lines, CMT167 and LLC; EML4/ALK cell lines, EA1 and EA2). Statistical analysis was performed on (C) using a one-way ANOVA. For A–C, data are from four replicates performed over three independent experiments. For C, vehicle controls were uniformly <0.5% MHC II+for each of the cell lines and were not included on the figure. (D–H) Human NSCLC lines (H1299, H1650, H460) were treated with vehicle or 100 ng/mL IFNγ for 48 hours, and analyzed by flow cytometry for cell surface expression of (D–E) PD-L1, (F–G) MHC I and (H) MHC II, quantifying either the MFI (D, F) or percentage of HLA-DR (ie, MHC II)+events (H). Representative histograms for PD-L1 and MHC I are shown in panels E, G, where unfilled black histograms are vehicle, and the filled histograms show cells treated with IFNγ. Statistical analysis shown in panels D, F and H used an unpaired t-test to determine statistical significance between vehicle and IFNγ treated samples (statistical significance identified by *p<0.05). All MFI data are x10^6^. Data are from three independent experiments (D–H). All flow cytometry data were gated on viable, singlets. Graphs show mean±SEM. ANOVA, analysis of variance; IFNγ, interferon gamma; LLC, Lewis lung carcinoma; SSC, side scatter.

We next evaluated IFNγ responsiveness and MHC II induction across additional lung cancer lines, comparing murine KRAS mutant (CMT167, LLC) and EML4-ALK fusion (EA1, EA2) cell lines.^14 15^ NSCLC lines were subjected to IFNγ stimulation and analyzed for the expression of IFNγ-inducible cell surface proteins by flow cytometry (figure 2). All four NSCLC lines showed uniform IFNγ-induced expression of PD-L1 and MHC class I proteins on the cell surface by 48 hours post-IFNγ stimulation, with 100% of each cell line expressing these proteins and a significant increase in median fluorescent intensity (figure 2A, B, online supplementary figure 1). In contrast, there was wide variation in the induction of cell surface MHC class II expression, within and between cell lines where: (1) CMT167 and EA1 cells induced MHC II in a subset of cells, in contrast to (2) LLC and EA2 cells which had negligible MHC class II expression under either basal or IFNγ stimulated conditions (figure 2C, online supplementary figure 2). Because MHC II is induced in only a fraction (~10%) of CMT167 and EA1 cells, in contrast to the uniform protein induction of PD-L1 and MHC I, the data in figure 2C are presented as the frequency of MHC II positive cells following IFNγ stimulation. The frequency of MHC II+events under baseline conditions was always <0.5% (data not shown); changes in MHC II MFI were obscured by the dominant fraction of MHC II negative events (online supplementary figure 2B).

10.1136/jitc-2019-000441.supp2 Supplementary data

IFNγ induction of cell surface proteins was also evaluated in a panel of human NSCLC cell lines. Three of three human NSCLC cell lines showed IFNγ-dependent induction of PD-L1 and MHC class I (HLA-A, B, C), with cells uniformly inducing these proteins (figure 2D–G). In contrast, there was variable IFNγ-dependent MHC II induction, with MHC II (HLA-DR) induced in a subset of H1650 and H460 cells, with no induction in the H1299 cell line (figure 2H). A larger panel of human NSCLC lines stimulated with IFNγ for 72 hours showed PD-L1, MHC I induction across the majority of cell lines, with MHC II (HLA-DR and HLA-DQ) again induced in a subset of cell lines (online supplementary figure 3).

**Figure 3:**
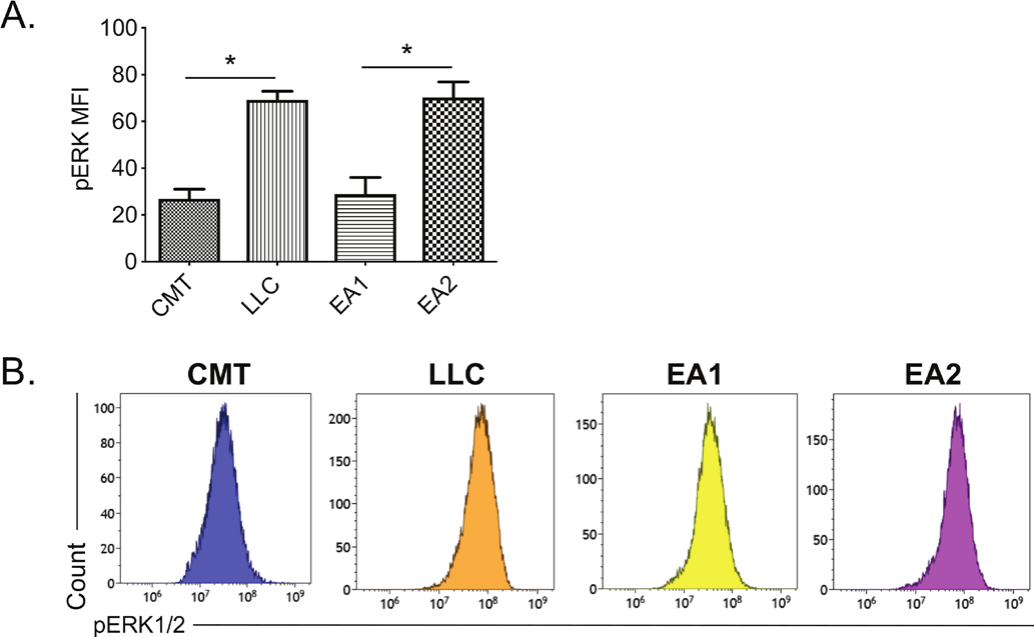
Correlation between MHC II induction and basal phosphorylated ERK1/2 levels in murine NSCLC cells. Mouse NSCLC lines were cultured for 48 hours, and analyzed for basal levels of intracellular pERK1/2 by flow cytometric analysis. (A) Relative pERK1/2 levels in four murine NSCLC cell lines, including (B) representative histograms. Data are from two independent experiments, with n=3 replicates total. Statistical comparisons of pERK levels were focused on comparison of oncogene-matched cancer cell lines; pERK levels were not significantly different between CMT167 and EA1 cells. MFI is x10^6^. Graphs show mean±SEM. Flow cytometry data analyzed singlets with MFI data visualized x10^6^ values. LLC, Lewis lung carcinoma; MFI, median fluorescent intensity; NSCLC, non-small cell lung cancer.

We next sought to understand mechanisms that restrain MHC II induction within and between NSCLC lines. Curiously, basal pERK1/2 levels were inversely correlated with high MHC II inducibility when we compared oncogene-matched NSCLC cell lines that had high vs low MHC II induction (ie, CMT167 vs LLC, EA1 vs EA2) (comparing figures 2C and 3). This inverse relationship between MHC II induction and basal pERK levels suggested that RAS-RAF-MEK-ERK signaling may impair MHC II induction in NSCLC cells.

**Figure 4:**
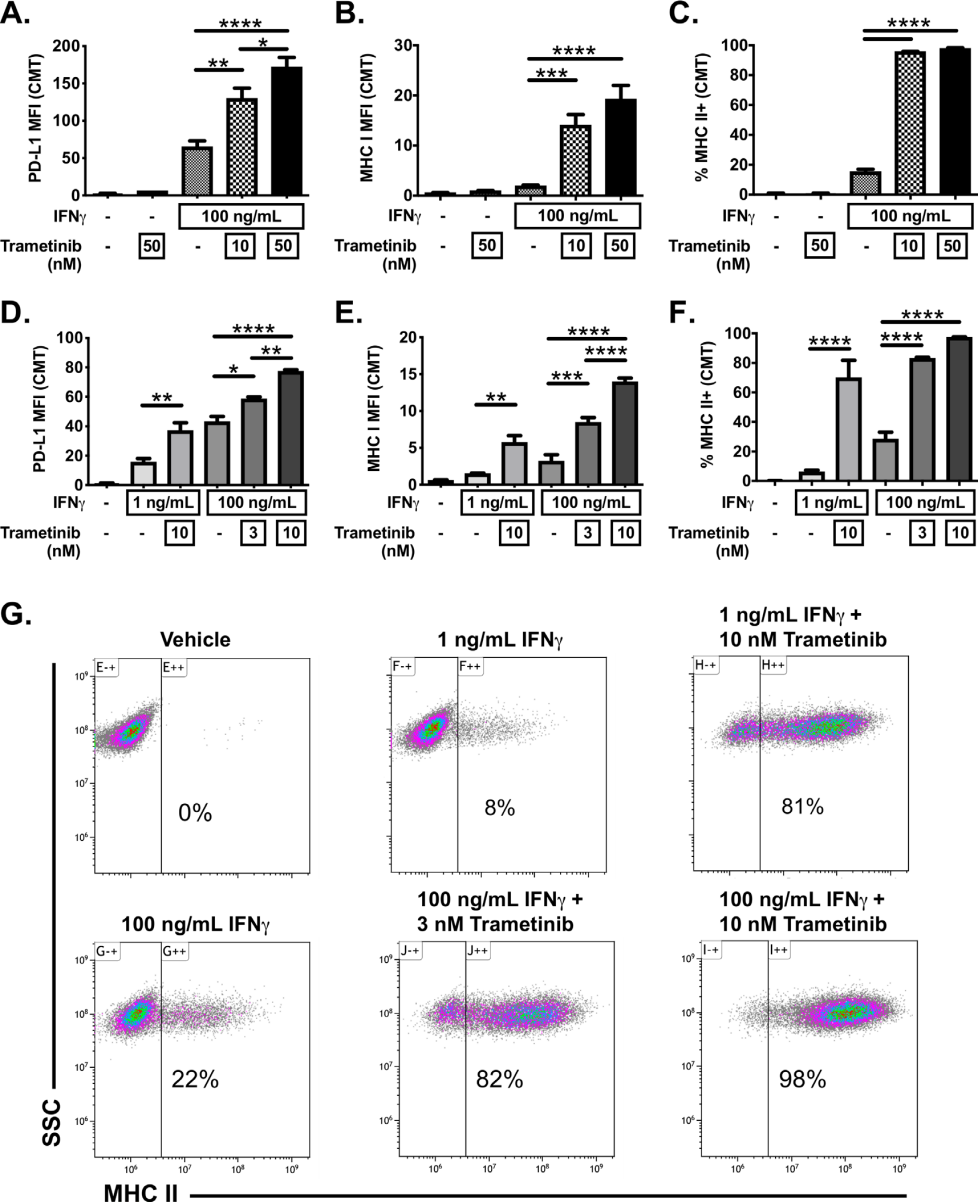
Trametinib enhances responsiveness to IFNγ in CMT167 cells. CMT167 cells were treated with vehicle or IFNγ, in the presence or absence of the MEK inhibitor, trametinib, at indicated concentrations for 48 hours. The cells were then collected, stained with the indicated antibody and analyzed using multiparameter flow cytometry for cell surface expression of (A, D) PD-L1 or (B, E) MHC I, defined by median fluorescent intensity (MFI) and (C, F, G) MHC II, defined by the frequency of MHC II positive events. (A–C) Depict studies where cells were either vehicle treated or stimulated with IFNγ (100 ng/mL), with data in (D–G) further quantifying the effect of varying concentrations of either IFNγ or trametinib on protein expression. (G) Representative flow cytometry dot plots showing the percentage of MHC II+events, defined relative to side scatter (SSC). All flow cytometry data were gated on viable, singlets with MFI as x10^6^ values. Data are from three independent experiments. Statistical analysis was done comparing the effect of trametinib on IFNγ induction using one-way ANOVA subjected to Sidak’s multiple comparisons test, with statistical significance indicated as follows: *p<0.05; **p<0.01; ***p<0.001; ****p<0.0001. Graphs show mean±SEM. ANOVA, analysis of variance; IFNγ, interferon gamma.

To determine if modulation of MEK/ERK signaling influences MHC II inducibility, or more generally IFNγ responsiveness, CMT167 cells were treated with IFNγ in the presence or absence of the MEK inhibitor (MEKi) trametinib (figure 4). As expected, IFNγ treatment of CMT167 cells induced PD-L1, MHC I and MHC II (figure 4A–C). Trametinib treatment alone was insufficient to induce these proteins (figure 4A–C). In contrast, simultaneous treatment of cells with IFNγ and trametinib enhanced PD-L1 expression (~2 fold) and MHC I expression (~7 fold) compared with IFNγ treatment alone, showing a dose-dependent enhancement (figure 4A–B). IFNγ plus trametinib further resulted in a dramatic increase in the frequency of MHC II expressing cells from ~20% (after IFNγ treatment) to nearly 100% of CMT167 cells (after combinatorial treatment with IFNγ and trametinib) (figure 4C). Trametinib enhanced IFNγ-inducible gene expression across a range of doses (from 3 to 50 nM) and following IFNγ stimulation across a range of concentrations (from 1 to 100 ng/mL IFNγ) (figure 4D–G). Note that in humans, at the Food and Drug Administration (FDA)-approved dose of 2 mg daily, the maximum serum concentration of trametinib is 36 nM^32^ suggesting that the concentrations used are clinically relevant. These data demonstrate that a MEKi is capable of potently enhancing IFNγ-inducible gene expression, promoting homogeneous induction of MHC II expression in CMT167 cells.

**Figure 5:**
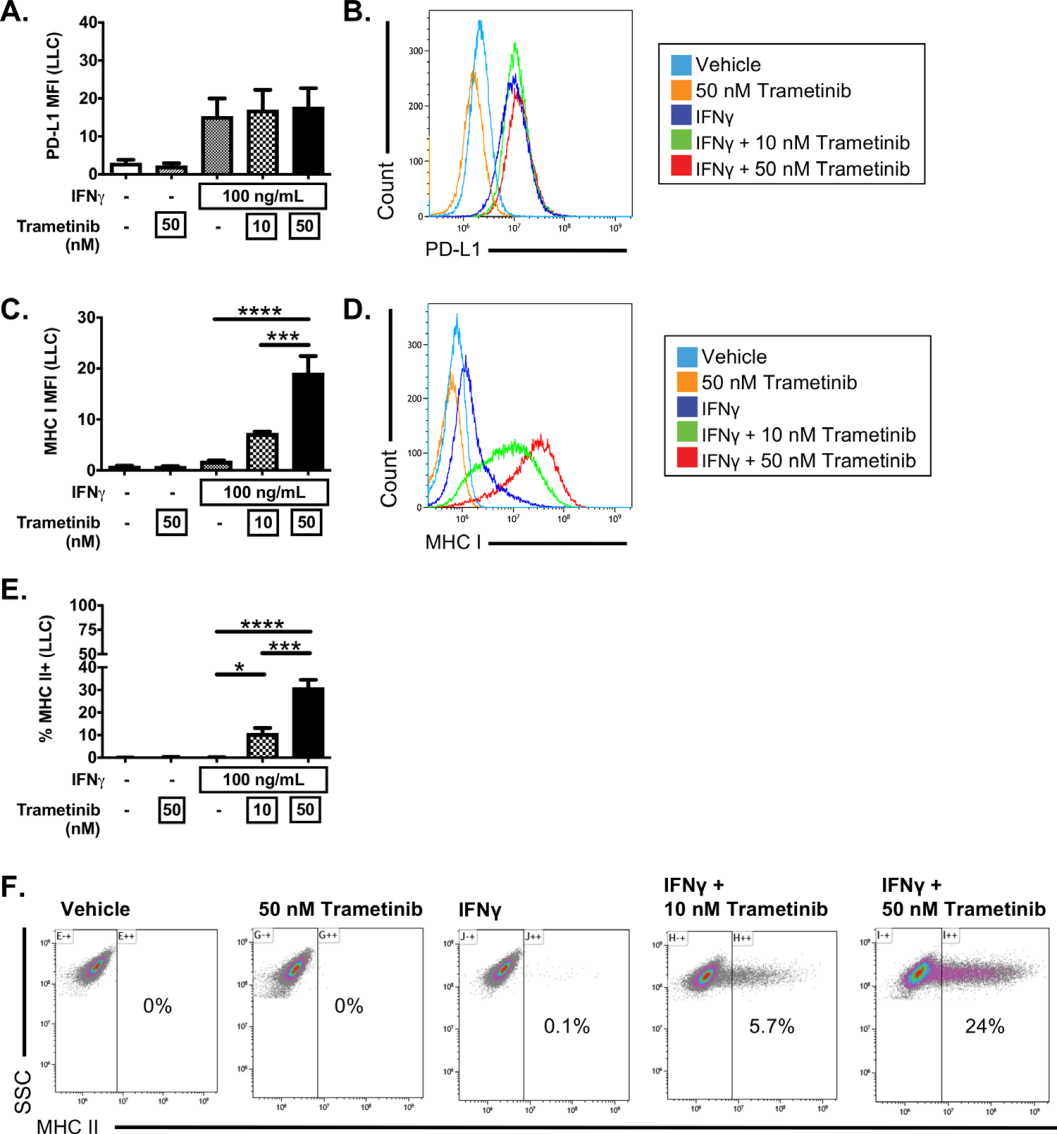
Trametinib enhances responsiveness to IFNγ in LLC cells. LLC cells were treated with vehicle or IFNγ (100 ng/mL), in the presence or absence of the MEK inhibitor, trametinib, at the indicated concentrations for 48 hours. Cells were then harvested and analyzed by flow cytometry for (A, B) PD-L1, (C, D) MHC I or (E, F) MHC II expression. Data depict median fluorescent intensity (MFI) with representative histograms to the right (B, D) or the frequency of MHC II+events with representative dot plots (E, F). Data are from three independent experiments. All flow cytometry data were gated on viable, singlets with MFI as x10^6^ values. Statistical analysis was done comparing the effect of trametinib on IFNγ induction using one-way ANOVA subjected to Sidak’s multiple comparisons test, with statistical significance indicated as follows: *p<0.05; **p<0.01; ***p<0.001; ****p<0.0001. Graphs show mean±SEM. ANOVA, analysis of variance; IFNγ, interferon gamma.

We next analyzed the impact of MEKi inhibition in LLC cells, a cell line that failed to induce MHC II expression after IFNγ stimulation. Treatment of LLC cells with trametinib in the absence of IFNγ induce a marked morphologic change in LLC cells (online supplementary figure 4), but failed to induce PD-L1, MHC I or MHC II (figure 5). Similar to CMT167 cells, combinatorial treatment with IFNγ and trametinib in LLC cells altered the IFNγ response. While trametinib did not further enhance IFNγ-dependent PD-L1 induction in LLC cells (figure 5A, B), cells treated with IFNγ plus trametinib had a dose-dependent increase in MHC I expression (figure 5C, D). Furthermore, IFNγ and trametinib cotreatment resulted in a marked induction of MHC II in a subset of LLC cells not seen in any other condition (figure 5E, F). Trametinib enhanced IFNγ induction of MHC II at low and high concentrations of IFNγ (from 1 to 100 ng/mL, online supplementary figure 5). To verify that this effect was due to MEK inhibition, and not a trametinib-specific effect, we also tested cobimetinib, an MEKi that has higher selectivity for MEK1 relative to MEK2, in contrast to trametinib which inhibits both MEK1 and MEK2.^33^ Combinatorial treatment of LLC cells with IFNγ and cobimetinib resulted in both enhanced MHC I expression on a per-cell basis and an increased frequency of MHC II expressing LLC cells (online supplementary figure 6). These data indicate that MEK inhibition at the time of IFNγ treatment has the capacity to significantly enhance IFNγ induction of both MHC I and MHC II expression, and further suggests that MEK signaling actively constrains MHC I and MHC II expression in response to IFNγ treatment.

Trametinib was further tested for its ability to modulate IFNγ responsiveness in human NSCLC cell lines. H1299 cells treated with IFNγ and trametinib had increased PD-L1 and MHC I expression relative to cells treated with IFNγ alone, though these cells failed to express MHC II under any conditions (online supplementary figure 7A). In contrast, IFNγ plus trametinib treatment did not enhance IFNγ-inducible PD-L1 or MHC I expression in either the H1650 or H460 cell lines, but this combinatorial treatment did increase the percentage of cells expressing MHC II (online supplementary figure 7B–C). Trametinib also substantially enhanced IFNγ-induced MHC II expression in the human NSCLC cell lines A549 and H358 (online supplementary figure 8). These data indicate that trametinib can enhance IFNγ responsiveness in human lung cancer cell lines, although with variable impact on IFNγ inducible targets.

**Figure 6:**
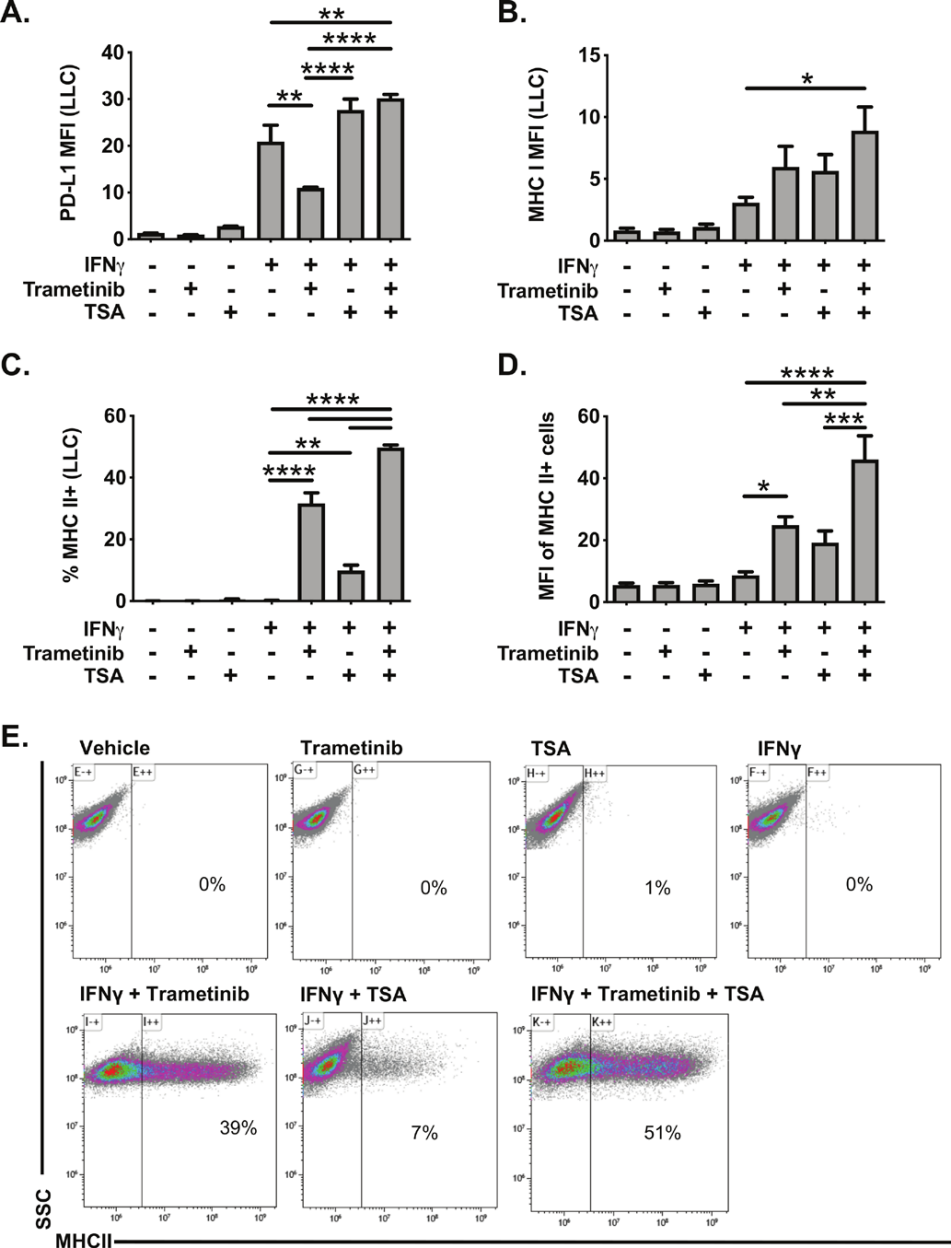
Trichostatin A and trametinib cotreatment leads to increased expression of interferon responsive proteins in LLC cells. LLC cells were pretreated for 24 hours with vehicle, trametinib (10 nM) and/or trichostatin A (TSA, 50 nM). The following day, media was changed, and cells were treated with vehicle, trametinib, TSA, and/or IFNγ for an additional 48 hours. Cells were then analyzed for cell surface expression of (A) PD-L1, (B) MHC I or (C–D) MHC II by flow cytometry. Data in panels A–D are from three independent experiments (mean±SEM), with representative density plots in panel E showing effect of various treatments on MHC II expression. All flow cytometry data were gated on viable, singlets with MFI as x10^6^ values. Statistical analysis was done comparing the effects of trametinib and TSA on IFNγ induction using one-way ANOVA subjected to Sidak’s multiple comparisons test, with statistical significance indicated as follows: *p<0.05; **p<0.01; ***p<0.001; ****p<0.0001. ANOVA, analysis of variance; IFNγ, interferon gamma; LLC, Lewis lung carcinoma; MFI, median fluorescent intensity; SSC, side scatter; TSA, trichostatin A.

Histone deacetylase inhibitors (HDACi) are another class of molecules that have been reported to promote MHC II expression in cancer cells.^34 35^ We, therefore, tested the impact of the HDACi TSA on IFNγ responsiveness in LLC cells. TSA treatment had minimal effect on PD-L1 or MHC I expression, alone or in combination with IFNγ (figure 6A–B). In contrast, TSA administration with IFNγ modestly increased the frequency of MHC II expressing LLC cells (figure 6C). TSA administration with IFNγ also enhanced expression of the MHC II pathway in the human KRAS mutant cell lines A549 and H358, measured by increased expression of CIITA mRNA expression and increased MHC II cell surface protein expression (online supplementary figure 9). While TSA was less effective than trametinib in inducing MHC II expression in LLC cells, treatment with TSA and trametinib was additive, further promoting the frequency, and expression, of MHC II in LLC cells (figure 6C–E). These data indicate that HDACs function to limit MHC II expression in the context of IFNγ stimulation, and that HDACs and MEK signaling both restrict MHC II expression.

Given that combinatorial treatment with an HDACi and MEKi only induced MHC II in a subset of LLC cells, we tested the impact of STAT3 and CDK4/6 inhibitors as additional candidate regulators of MHC II expression. Notably, both STAT3^36^ and CDK 4/6^37^ have been identified as regulators of the anti-tumor response, with the potential to regulate MHC II expression. Neither napabucasin (a STAT3 inhibitor) nor palbociclib (a CDK4/6 inhibitor) treatment enhanced IFNγ-dependent MHC II expression in LLC cells, indicating that these pathways, at least in isolation, did not constrain MHC II expression (online supplementary figure 10).

**Figure 7:**
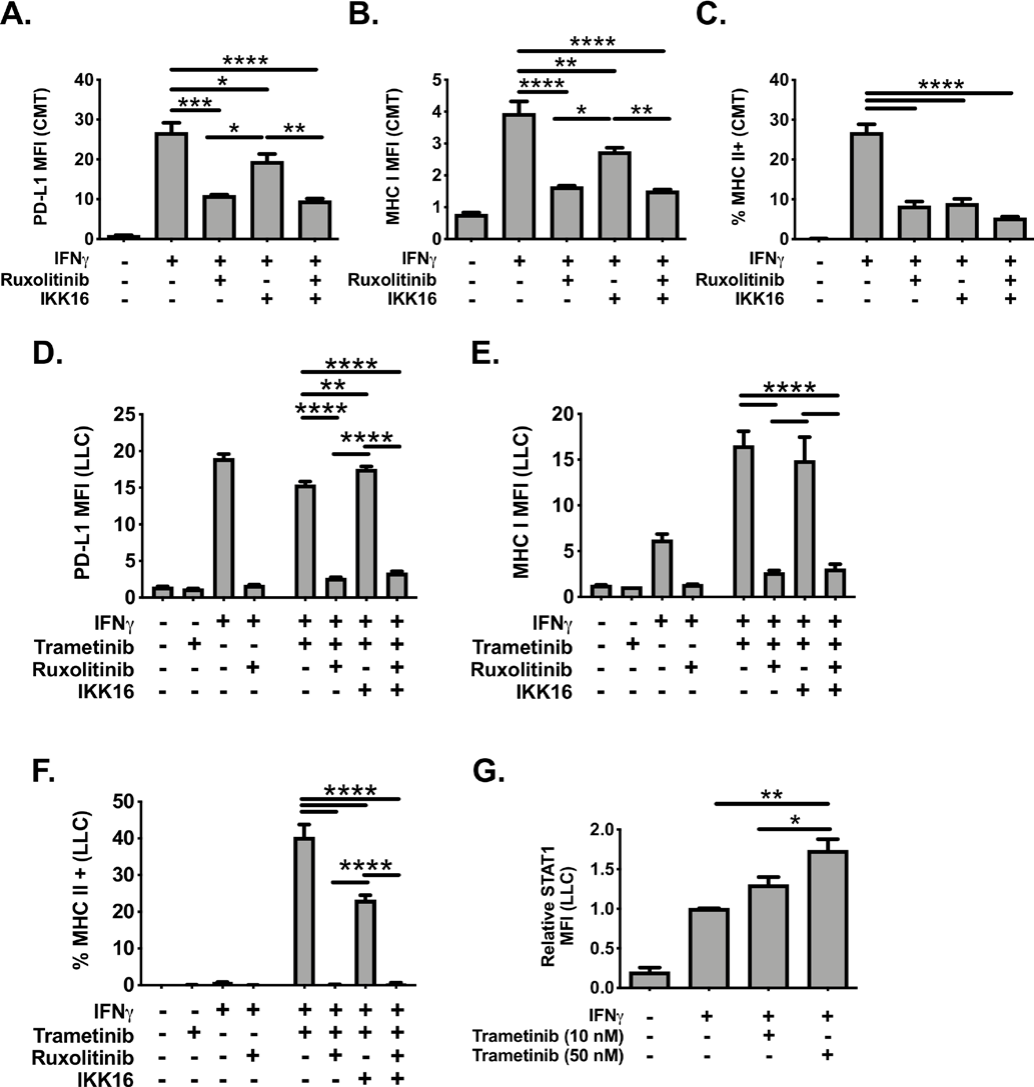
Analysis of the contribution of JAK/STAT and NF-kB dependent signals on IFNγ responsiveness in NSCLC lines. CMT167 (A–C) or LLC (D–F) cells were cultured for 48 hours with IFNγ (100 ng/mL), in the presence or absence of various inhibitors. (A–C) CMT167 cells were cultured for 48 hours with IFN gamma (100 ng/mL), the JAK/STAT inhibitor ruxolitinib (1 µM), and/or the IκB kinase (IKK) inhibitor IKK-16 (500 nM). Cells were then analyzed by flow cytometry for surface protein expression as indicated. (D–F) LLC cells were cultured for 48 hours with IFNγ (100 ng/mL), trametinib (10 nM), ruxolitinib (1 µM) and/or IKK-16 (500 nM). Cells were analyzed by flow cytometry for cell surface expression of (A, D) PD-L1, (B, E) MHC I and (C, F) MHC II. (G) LLC cells were cultured for 48 hours with interferon gamma (100 ng/mL) and/or trametinib at indicated concentrations. Cells were then collected, fixed, permeabilized and analyzed for intracellular total STAT1 levels. All flow cytometry data were gated on viable singlets, except G in which cells were fixed and gated on singlets. Two independent experiments were performed, with n=3 replicates total for each variable analyzed. Statistical analysis was done comparing the effect of inhibitors on induction by IFNγ (A–C), by IFNγ plus trametinib (D–F), or trametinib (panel G) using one-way ANOVA subjected to Sidak’s multiple comparisons test, with statistical significance indicated as follows: *p<0.05; **p<0.01; ***p<0.001; ****p<0.0001. Graphs show mean±SEM. ANOVA, analysis of variance; IFNγ, interferon gamma; LLC, Lewis lung carcinoma; MFI, median fluorescent intensity.

We next explored the mechanisms promoting IFNγ responsiveness in either CMT167 cells treated with IFNγ, or with LLC cells treated with IFNγ in the presence or absence of trametinib. For these studies, we focused on the role of the janus kinase (JAK)-signal transducer and activator of transcription (STAT) pathway (defined by use of the JAK/STAT inhibitor, ruxolitinib, at the clinically relevant concentration of 1 µM^38^) and the role of NF-kB (defined by use of the IKK inhibitor, IKK-16). CMT167 cells treated with IFNγ showed inducible PD-L1, MHC I and MHC II expression, which was inhibited by ruxolitinib and to a lesser extent IKK-16 (figure 7A–C). Among these two pathways, the JAK/STAT pathway played a dominant role, with combinatorial inhibition by IKK-16 and ruxolitinib affording no additional benefit beyond ruxolitinib treatment alone (figure 7A–C). LLC cells treated with IFNγ with or without trametinib showed the anticipated induction of PD-L1, MHC I and MHC II. For PD-L1 and MHC I, ruxolitinib treatment, but not IKK-16, impaired induction generated by IFNγ and trametinib cotreatment (figure 7D–E). For MHC II, ruxolitinib completely inhibited IFNγ/trametinib MHC II induction whereas IKK-16 treatment resulted in ~50% reduction in the frequency of MHC II expressing cells (figure 7F). These data indicate that the NF-κB signaling pathway contributes to maximal IFNγ-induced MHC II expression, but is not involved in either PD-L1 or MHC I induction in LLC cells. In contrast, JAK/STAT signaling is involved in IFNγ-induced expression of MHC I, MHC II and PD-L1 in both CMT167 and LLC cells. LLC cells cotreated with IFNγ and trametinib had increased levels of STAT1 (figure 7G), emphasizing the critical role of STAT1 in promoting IFNγ signaling under both basal and MEK inhibition conditions.

## Discussion

Immunotherapy has led to dramatic improvements in outcomes in a subset of patients with NSCLC in recent years.^3^ However, the majority of patients treated with PD-1 immunotherapy fail to respond. Thus, extensive research efforts are underway to identify methods to improve response rates to immunotherapy. It has recently been demonstrated that tumors with an IFNγ responsive gene signature have improved outcomes when treated with PD-1/PD-L1-targeted immunotherapy.^5^

In this manuscript, we sought to investigate how IFNγ responsiveness is regulated in a panel of mouse and human NSCLC lines, with a primary focus on the CMT167 and LLC mouse lung tumor lines that differ in their response to PD-1/PD-L1-targeted blockade.^11^ LLC cells are resistant to checkpoint blockade with PD-1/PD-L1 antibodies^16^ and have a relatively high basal pERK levels (figure 3); conversely, CMT167 cells are sensitive to checkpoint blockade^16^ and have low basal pERK levels. It is thus possible that ERK/MEK signaling may affect PD-1/PD-L1 responsiveness via regulation of IFN signaling. This hypothesis is consistent with our studies demonstrating that the MEKi trametinib and cobimetinib lead to increased induction of MHC I and MHC II in response to IFNγ stimulation (figure 5, online supplementary figure 6).

We demonstrate that CMT167 cells are capable of robustly responding to IFNγ, resulting in the induction of PD-L1, MHC I and MHC II. In contrast, LLC cells had a more focused response to IFNγ, resulting in the induction of PD-L1 and MHC I but not MHC II. IFNγ-dependent induction of PD-L1 and MHC I was observed across all mouse and human NSCLC lines that were analyzed, indicating that the cell lines tested did not have a general defect in IFNγ signaling. In contrast, MHC II induction in response to IFNγ was only observed in a subset of cell lines. While the failure of MHC II induction could result from a defect in the MHC II pathway, MHC II expression could be induced to at least some extent by combinatorial treatments, again suggesting that there was not a genetic defect in the MHC II pathway. These data emphasize that the IFNγ and MHC II pathways are not defective in the cell lines tested here, instead implicating an active mechanism constraining MHC II induction. Our studies sought to define mechanism(s) of MHC II regulation in NSCLC cell lines, with a focus on MEK-ERK and HDAC-dependent pathways.

The MEK-ERK pathway has previously been implicated in regulating the expression of MHC I and MHC II.^39^ Here, we found that cells stimulated with IFNγ in the presence of a MEKi (either trametinib or cobimetinib) showed enhanced induction of MHC I and MHC II. Notably, the impact of MEK inhibition was equivalent to (for MHC I) or larger (for MHC II) than the effect of HDAC inhibition alone. These data emphasize that MEK signaling actively constrains IFNγ inducible gene expression, and further suggest that MEK signaling either directly modifies IFNγ dependent signal transduction (eg, by altering STAT1 phosphorylation or post-translational modifications on the MHC II transactivator, CIITA^40^), or actively shapes local epigenetic modifications that are remodeled during inducible gene expression.^41^ Consistent with a broader impact of MEKi on inducible/inflammatory gene expression, a recent manuscript showed that multiple MEK and ERK inhibitors can enhance cancer cell line responsiveness to TNFα and IFNγ, a phenotype associated with increased NF-kB activity.^42^ While our data do not discriminate between a focused impact on IFNγ signaling or broader inducible gene expression, MEKi enhancement of IFNγ targets in our studies was critically dependent on JAK/STAT signaling, as revealed by use of the inhibitor ruxolitinib. How MEKi mechanistically enhance MHC II inducibility in NSCLC cell lines, and whether this is linked with HDAC function or activity, remains an important unanswered question at this time.

A primary focus of our current studies has been on the expression of MHC II, given its divergent regulation between cell lines regardless of oncogene driver. It is important to note that MHC II expression, and the broader MHC II processing and presentation pathway, is coordinately regulated by the MHC class II transactivator, CIITA,^43^ a regulatory network that is highly specific to induction of the MHC II pathway.^44^ It is well established that epigenetic regulation plays an important role in determining expression of MHC class II.^35 45^ Our studies using TSA, a pan-HDAC inhibitor, strongly implicate one or more HDACs as negative regulators of MHC II expression in NSCLC cell lines, consistent with previously published reports of TSA- and HDAC-dependent regulation of MHC II in other contexts.^46 47^ Which HDAC(s) regulate MHC II inducibility in NSCLC cell lines, and whether HDAC activity may be mechanistically linked to MEK-ERK signaling, remains unknown. Future research will benefit from the study of different classes of HDACi,^48^ with the goal to enhance IFNγ-dependent MHC II expression while minimizing alterations to additional transcriptional programs.

Based on the enhanced induction of MHC II following treatment with MEKi and HDACi, we postulate that cell-to-cell variation in these pathways may contribute to the variable induction of MHC II following IFNγ stimulation. Further, we postulate that differential responsiveness to IFNγ stimulation observed between NSCLC cell lines may result from inter-cell line variability in: (1) the baseline activation of MEK/ERK signaling pathways and (2) locus-specific variation in chromatin accessibility (eg, in the CIITA gene). Consistent with this model, MHC II is uniformly induced in CMT167 cells following IFNγ and MEKi cotreatment. In contrast, the failure to uniformly induce MHC II in LLC cells following simultaneous treatment with IFNγ, MEKi and HDACi strongly suggests that MEK-ERK signaling and HDACs actively restrain IFNγ-dependent signaling in some, but not all, NSCLC cell lines, and that there are additional, unidentified modulators of this pathway.

Our studies emphasize an important link between MEK and HDAC inhibitors and potential sensitivity of NSCLC cell lines to mechanisms of immune detection by regulating cancer cell-specific MHC II expression. It has previously been shown in triple negative breast cancer that alterations in RAS/MAPK signaling is associated with reduced tumor infiltrating lymphocytes. MEK inhibition led to increased MHC expression and was synergistic with anti-PD-1 immunotherapy in syngeneic models.^39^ Similarly, immunotherapy was synergistic with BRAF and MEK inhibition in preclinical melanoma models.^49^ However, the mechanistic basis for how MEK inhibition can promote immunotherapy success remains poorly understood, particularly in the context of NSCLC. Our studies strongly suggest that MEK inhibition enhances JAK-STAT signaling activation in response to IFNγ stimulation, with a particularly pronounced effect on the induction of MHC I and MHC II. We further postulate that the beneficial effects of MEK and HDAC inhibitors are mediated at least in part by enhancing cancer cell-intrinsic antigen processing and presentation pathways, potentially enabling CD8 and CD4 T cell recognition of cancer cells through increased MHC class I and II expression and MHC-dependent presentation of a larger, more diverse repertoire of tumor neoantigens.

## Conclusions

In total, our studies demonstrate that IFNγ signaling in NSCLC cell lines is actively restrained by HDAC-dependent epigenetic modifications and by MEK-ERK signaling. How these two pathways regulate MHC I and MHC II expression at the molecular level, and whether these pathways are mechanistically linked, remains an ongoing subject of investigation. Given that both HDACs and MEK-ERK signaling can be therapeutically targeted, our data emphasize the potential utility of HDAC and/or MEK inhibition in individuals with an IFNγ rich TME. The potential utility of these combinatorial treatments is further exemplified by recent studies showing the benefit of either HDAC inhibition or MEK inhibition in the context of PD-1/PD-L1 targeted immunotherapy.^50 51^ These findings support further study, both preclinically and clinically, of MEKi and their impact on tumor immunogenicity, particularly in patients undergoing immunotherapy-based treatment for NSCLC.
