# Supplementary material for: Cancer cell-intrinsic expression of MHC II in lung cancer cell lines is actively restricted by MEK/ERK signaling and epigenetic mechanisms
Source: J Immunother Cancer. 2020 Apr 19;8(1):e000441. doi: 10.1136/jitc-2019-000441 (PMC7204826; doi:10.1136/jitc-2019-000441)
Supplement: Supplementary data [file jitc-2019-000441supp001.pdf]

Supplemental Table 1. Antibody panel used for mass cytometric analysis of CMT and LLC NSCLC lines treated with IFNγ.

Tag	Target	Clone/Product	Surface/Intracellular	Clustering
^{89}Y	CD45	H130	Surface	Yes
^{141}Pr	pSHP2 [Y580]	D66F10	Intracellular	Yes
^{142}Nd	CD11c	N418	Surface	Yes
^{144}Nd	MHC I	28-14-8	Surface	Yes
^{145}Nd	PE-PD-L1 / Anti-PE	10F.9G2 (Tonbo) / PE-001	Surface	Yes
^{146}Nd	pEGFR [Y1068]	D7A5	Intracellular	Yes
^{148}Nd	CD11b	M1/70	Surface	Yes
^{150}Nd	pRb [S807/811]	J112-906	Intracellular	Yes
^{152}Sm	pAkt [S473]	D9E	Intracellular	Yes
^{153}Eu	pStat1 [Y701]	58D6	Intracellular	Yes
^{156}Gd	p-p38 [T180/Y182]	D3F9	Intracellular	Yes
^{158}Gd	pStat3 [Y705]	4/P-Stat3	Intracellular	Yes
^{159}Gd	RORgt	BD2	Intracellular	Yes
^{160}Gd	FITC-anti-CD122 / Anti-FITC	TM-β1 (Biolegend)/ FIT-22	Surface	Yes
^{162}Dy	Ly6C	HK1.4	Surface	Yes
^{164}Dy	IkBa	L35A5	Intracellular	Yes
^{165}Ho	Beta-catenin (active)	D13A1	Intracellular	Yes
^{167}Er	pERK 1/2 [T202/Y204]	D1314.4E	Intracellular	Yes
^{168}Er	Ki-67	B56	Intracellular	Yes
^{169}Tm	Ly-6A/E (Sca-1)	D7	Surface	Yes
^{170}Er	Biotin-PD-L2 / Anti-Biotin	TY25 (Biolegend) / 1D4/C5	Surface	Yes
^{171}Yb	CD44	IM7	Surface	Yes
^{172}Yb	pS6 [S235/S236]	N7-548	Intracellular	Yes
^{173}Yb	CD117 (ckit)	2B8	Surface	Yes
^{174}Yb	MHC class II	M5/114.15.2	Surface	Yes
^{175}Yb	pHistone H3 [S28]	HTA28	Intracellular	Yes
^{176}Yb	APC-anti-CD24 / Anti-APC	M1/69 (Biolegend) / APC003	Surface	Yes
^{195}Pt	Cisplatin	Cell-ID Cisplatin		No
^{191}Ir, ^{193}Ir	Intercalator	Cell-ID Intercalator-Ir		No
^{140}Ce ^{151}Eu ^{153}Eu ^{165}Ho ^{175}Lu	Normalization Beads			No

$^{102, 104, 105, 106, 108, 110}Pd$	Isotopic Barcodes	Fluidigm Cell-ID 20 Plex Palladium barcoding kit		No
