# Supplementary material for: Cancer cell-intrinsic expression of MHC II in lung cancer cell lines is actively restricted by MEK/ERK signaling and epigenetic mechanisms
Source: J Immunother Cancer. 2020 Apr 19;8(1):e000441. doi: 10.1136/jitc-2019-000441 (PMC7204826; doi:10.1136/jitc-2019-000441)
Supplement: Supplementary data [file jitc-2019-000441supp002.pdf]

Supplemental figures

"Cancer cell-intrinsic expression of MHC II in lung cancer cell lines is actively restricted by MEK/ERK signaling and epigenetic mechanisms"

Alexander J. Neuwelt[1], Abigail K. Kimball[2], Amber M. Johnson[3], Benjamin W. Arnold[3], Bonnie L. Bullock[3], Rachael E. Kaspar[3], Emily K. Kleczko[3], Jeff W. Kwak[3], Meng-Han Wu[3], Lynn E. Heasley[4,5], Robert C. Doebele[3], Howard Y. Li[6,7], Raphael A. Nemenoff[3]*, Eric T. Clambey[2]*

[1] Department of Medical Oncology, Hunter Holmes McGuire Veterans Affairs Medical Center, Richmond, VA 23249
[2] Department of Anesthesiology, [3]Department of Medicine, and [4]Department of Craniofacial Biology, University of Colorado Denver | Anschutz Medical Campus, Aurora, CO 80045
[5] Veterans Affairs Medical Center, Denver, CO, 80220
[6] Department of Internal Medicine, Division of Pulmonary Disease and Critical Care Medicine, Virginia Commonwealth University, Richmond, VA 23298
[7] Medical Service, Pulmonary Section, Hunter Holmes McGuire Veterans Affairs Medical Center, Richmond, VA 23249

* Co-corresponding authors. Address correspondence and reprint requests to Dr. Raphael Nemenoff (Raphael.Nemenoff@cuanschutz.edu) and Dr. Eric Clambey (Eric.Clambey@cuanschutz.edu).

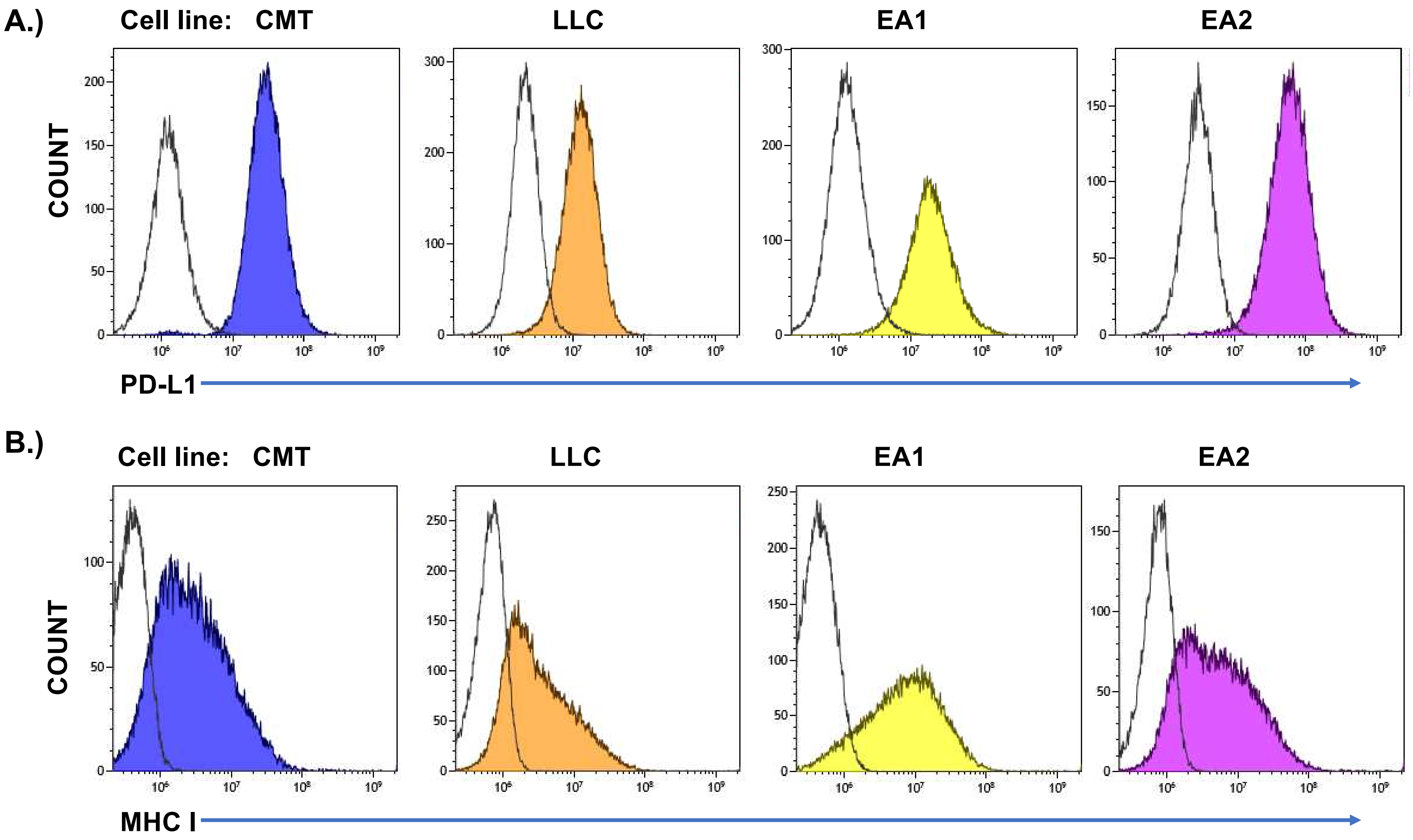
A.)
Cell line: CMT
LLC
EA1
EA2
COUNT
PD-L1
B.)
Cell line: CMT
LLC
EA1
EA2
COUNT
MHC I

Supplemental Figure 1: PD-L1 and MHC I expression are induced by interferon gamma in mouse NSCLC cell lines. Cultured cell lines (CMT, LLC, EA1 and EA2) were treated with vehicle or 100 ng/mL IFN gamma for 48 hours, and analyzed by flow cytometry for (A) PD-L1 and (B) MHC I. Representative histogram overlays are depicted for each cell line (column) comparing expression in vehicle treated (open, black lines) and interferon-gamma treated (shaded) cells. Data correspond to studies presented in Figure 2A-B.

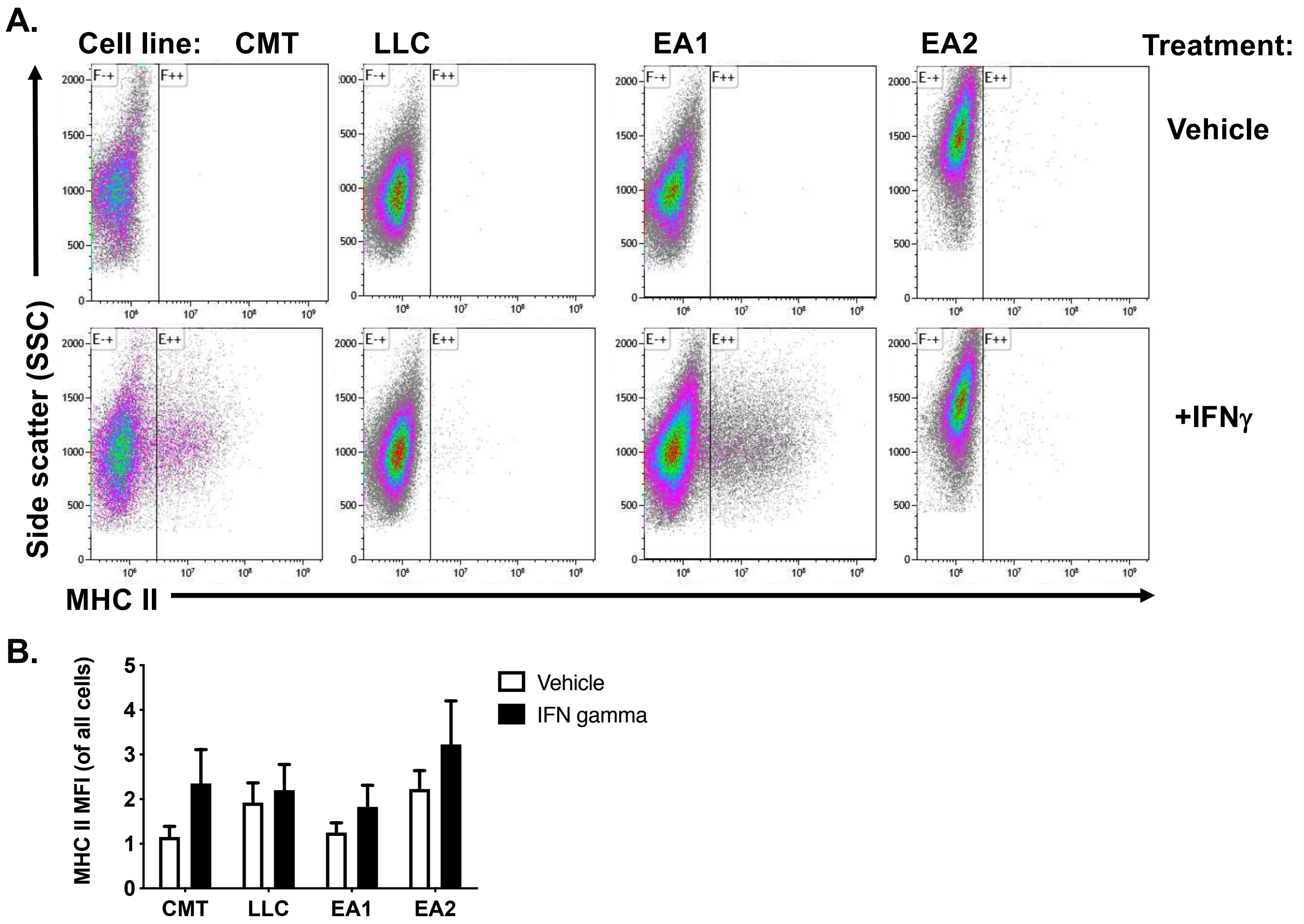
A.
Cell line:
CMT
LLC
EA1
EA2
Treatment:
Vehicle
+IFNγ
Side scatter (SSC)
MHC II
B.
MHC II MFI (of all cells)
Vehicle
IFN gamma
CMT
LLC
EA1
EA2

Supplemental Figure 2: MHC II is variably induced by interferon gamma in mouse NSCLC cell lines. (A) Cultured cell lines (CMT, LLC, EA1 and EA2) were treated with vehicle or 100 ng/mL IFN gamma for 48 hours, and analyzed by flow cytometry for surface MHC II. Representative density plots are shown, depicting side scatter (y axis) versus MHC II (x axis), comparing cells treated with vehicle (top) and IFN gamma (bottom). **(B)** Median fluorescent intensity of MHC II expression, based on MHC II expression across all cells. Data are from 4 replicates performed over 3 independent experiments. There was no statistical significance in MHC II MFI, using an unpaired t-test to compare MHC II MFI between vehicle and interferon-gamma treated cells. Data correspond to studies presented in Figure 2C.

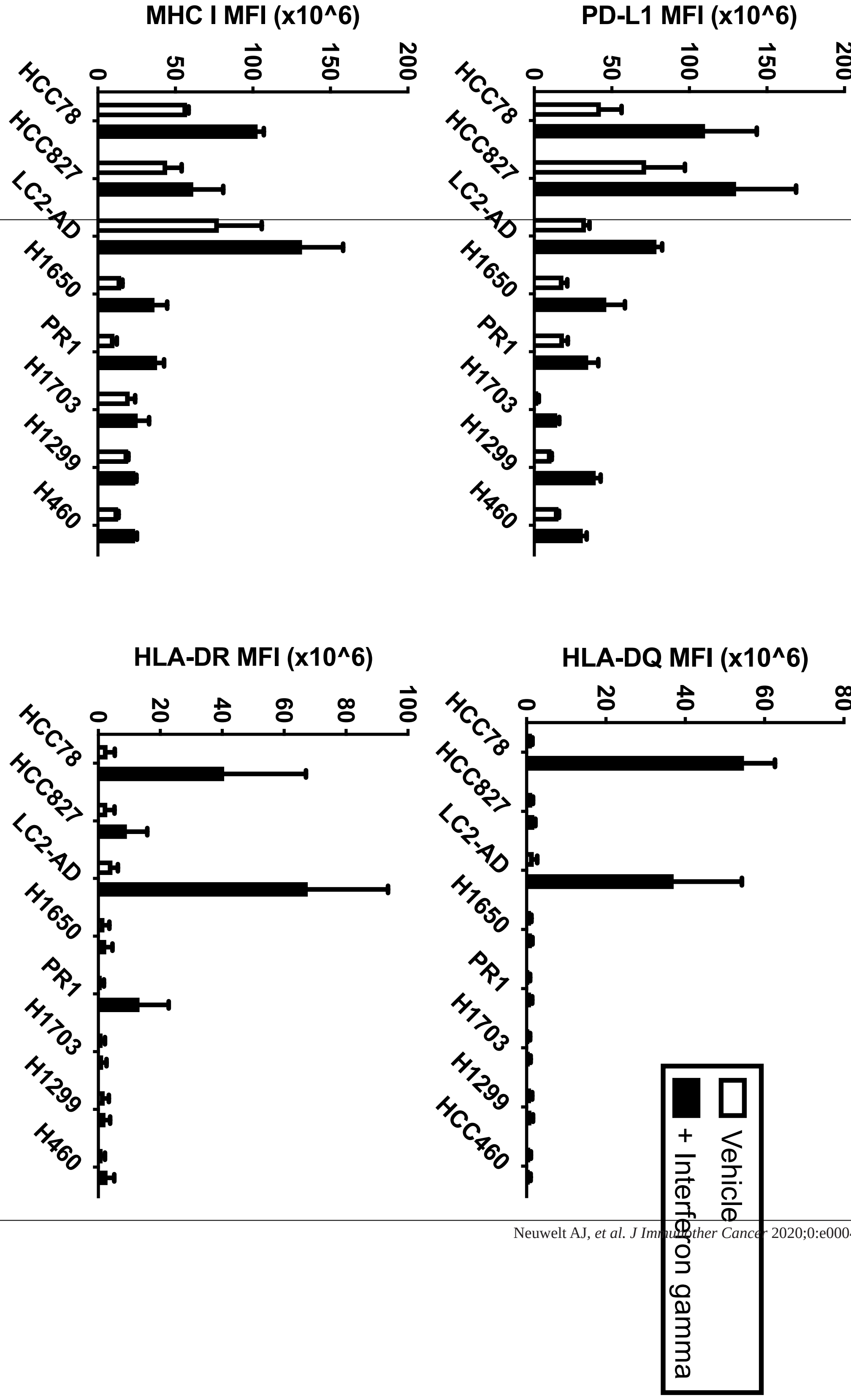
PD-L1 MFI (x10^6)
MHC I MFI (x10^6)
HLA-DQ MFI (x10^6)
HLA-DR MFI (x10^6)
HCC78
HCC827
LC2-AD
H1650
PR1
H1703
H1299
H460
HCC460
Vehicle
+ Interferon gamma

Supplemental Figure 3: Cell surface expression of PD-L1, MHC I, and MHC II proteins in human NSCLC cell lines. Human non-small cell lung cancer cell lines were incubated for 72 hours in vitro with either vehicle or interferon gamma (100 ng/mL). Cells were collected, with cell surface expression of PD-L1, MHC I, HLA-DQ and HLA-DR measured by flow cytometry. Cells were gated on live singlets. Data depict n=2 experiments, plotting mean +/- range.

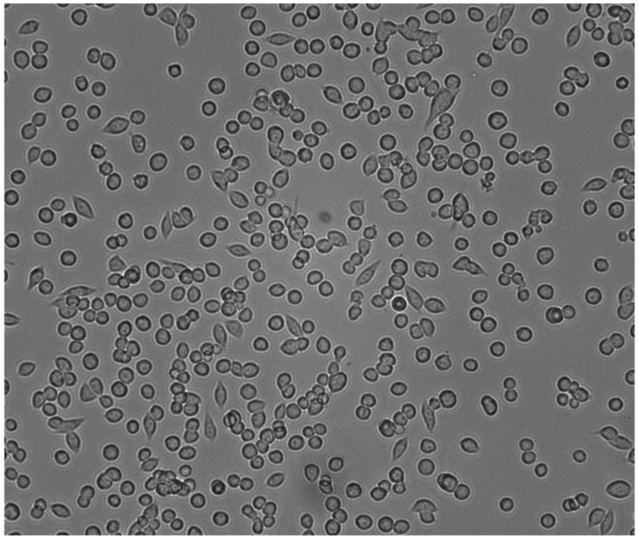

LLC, Vehicle treated

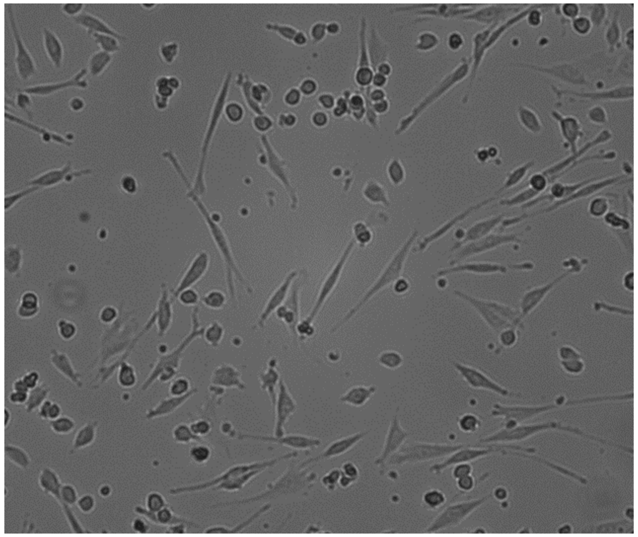

LLC, Trametinib treated

Supplemental Figure 4: Trametinib treatment results in a morphologic change in LLC cells. LLC cells were treated for 48 hours with 100 nM trametinib, and morphologic changes were documented.

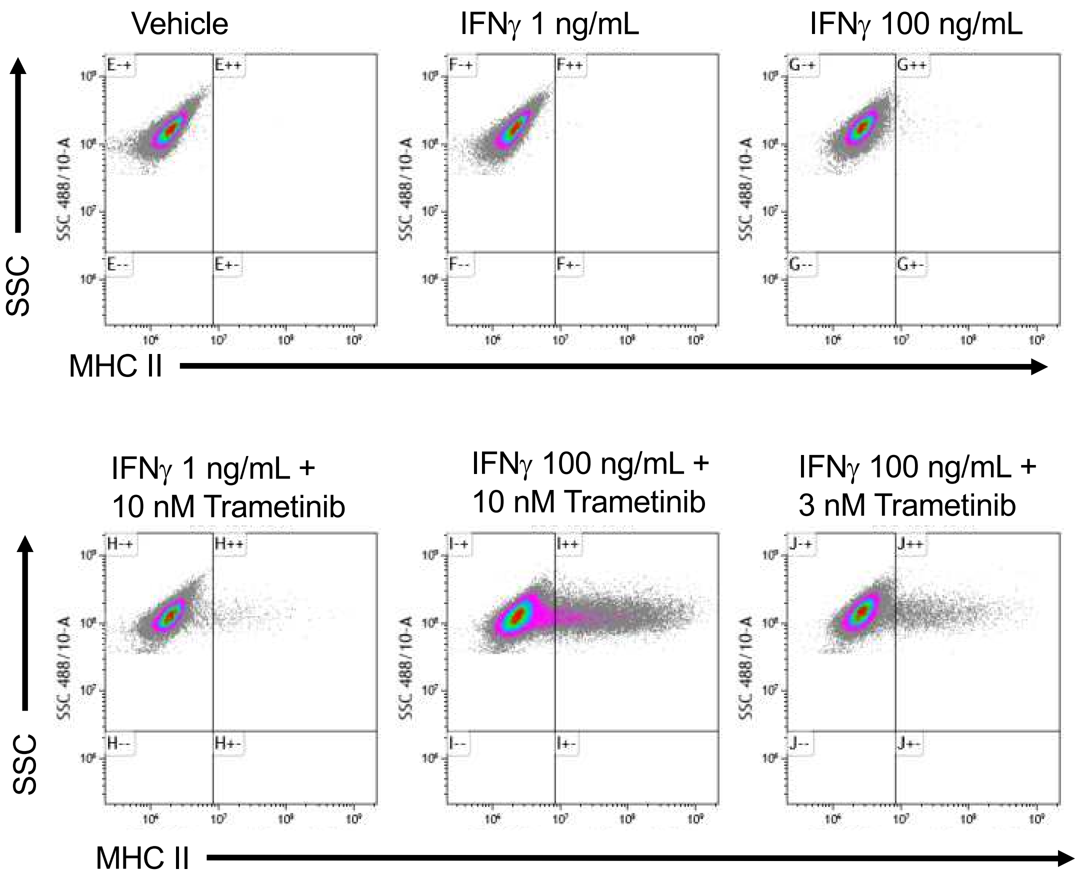
Vehicle
IFNγ 1 ng/mL
IFNγ 100 ng/mL
SSC
MHC II
IFNγ 1 ng/mL + 10 nM Trametinib
IFNγ 100 ng/mL + 10 nM Trametinib
IFNγ 100 ng/mL + 3 nM Trametinib
SSC
MHC II

Supplemental Figure 5: Induction of MHC II in LLC cells treated with interferon gamma and trametinib. LLC cells were treated for 48 hours with vehicle, or with varying doses of interferon gamma and/or trametinib, as indicated. The cells were then collected and analyzed for surface MHC II expression by flow cytometry. Cells gated on live singlets.

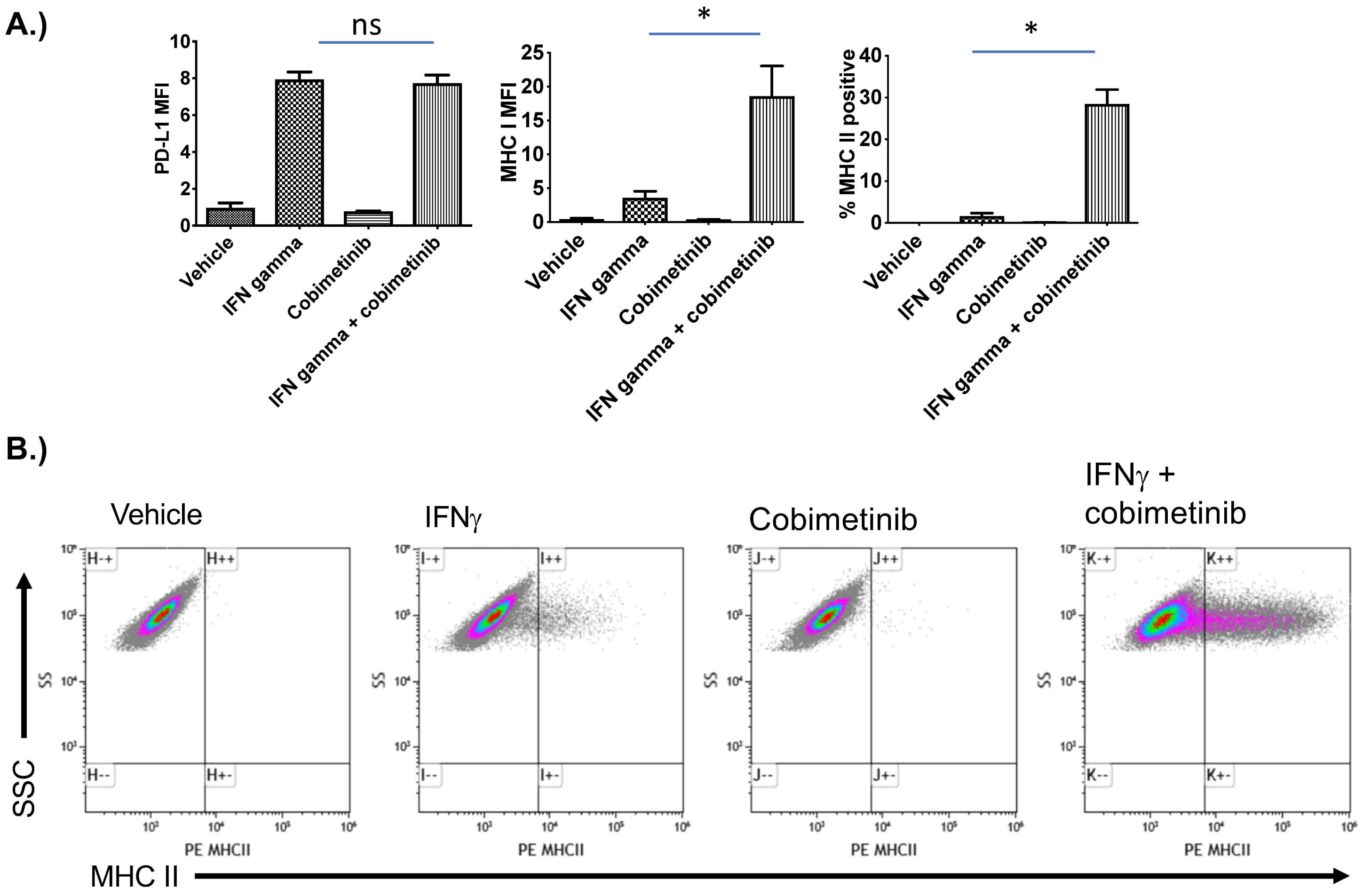
A.)
ns
*
*
PD-L1 MFI
MHC I MFI
% MHC II positive
Vehicle
IFN gamma
Cobimetinib
IFN gamma + cobimetinib
B.)
Vehicle
IFNγ
Cobimetinib
IFNγ + cobimetinib
SSC
MHC II
PE MHCII
SS
H-+
H++
H--
H+-
I-+
I++
I--
I+-
J-+
J++
J--
J+-
K-+
K++
K--
K+-

Supplemental Figure 6: Cobimetinib synergizes with interferon-gamma treatment to enhance MHC I and MHC II expression in LLC cells. LLC cells were treated with interferon gamma 100 ng/mL and/or cobimetinib (500 nM) for 48 hours, and then collected for analysis by flow cytometry. (A) Quantification of median fluorescent intensity (PD-L1, MHC I) or the percent positive events (MHC II, right plot). (B) Representative density plots showing cell surface expression of MHC II as a function of either interferon-gamma and/or cobimetinib treatment. Data depict results from three biologic replicates. Statistical analysis was performed using a one-way ANOVA with a Tukey post-test, * indicates significance $p < 0.05$. Events were gated for live singlets.

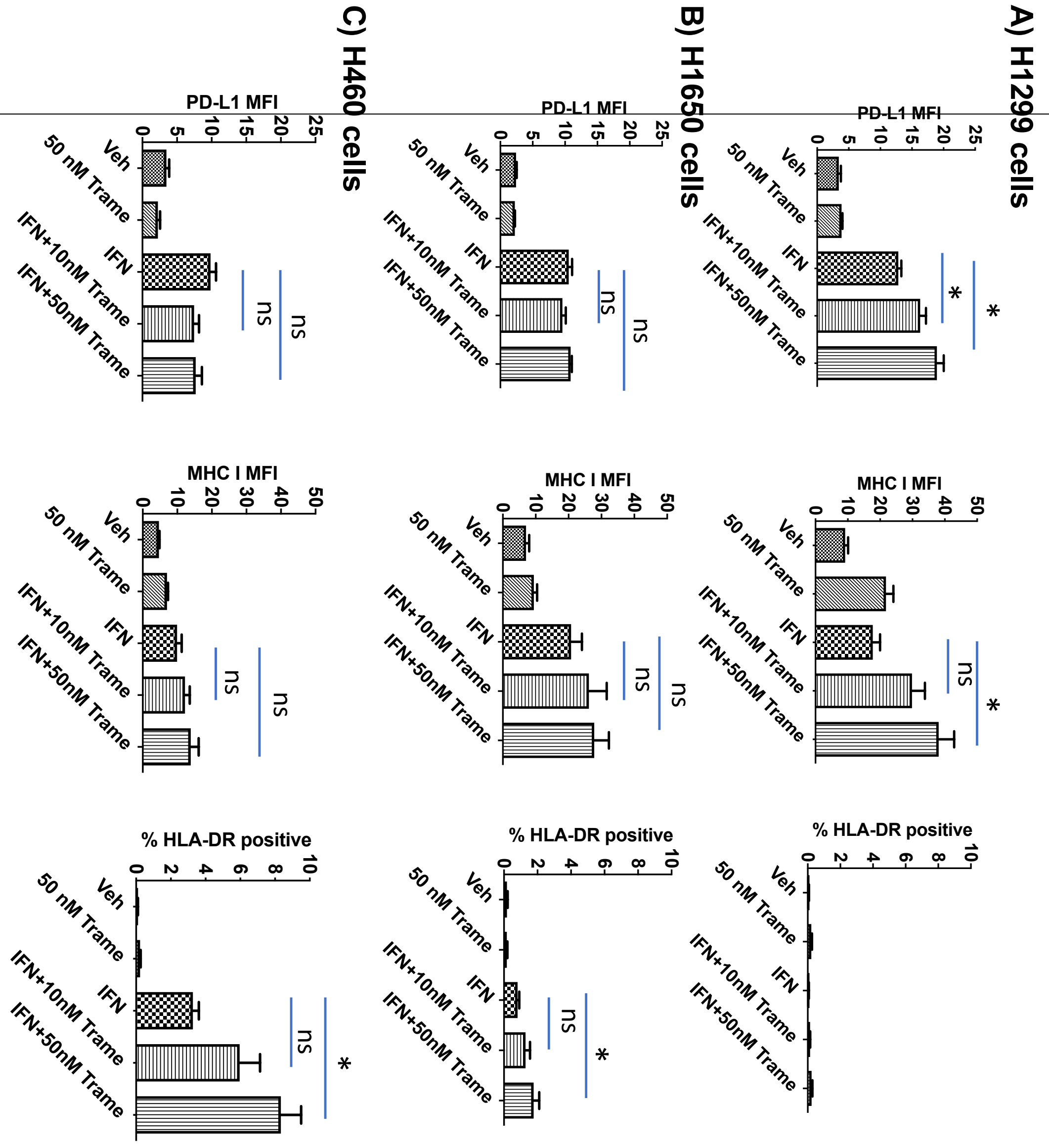
A) H1299 cells
B) H1650 cells
C) H460 cells
PD-L1 MFI
MHC I MFI
% HLA-DR positive
Veh
50 nM Trame
IFN
IFN+10nM Trame
IFN+50nM Trame
ns
*

Supplemental Figure 7: Trametinib has variable effects on interferon-responsive genes in human NSCLC lines. Human NSCLC lines (A, H1299), (B, H1650), and (C, H460) were treated for 48 hours with vehicle, interferon gamma (100 ng/mL) and/or trametinib at indicated concentration. Cells were then collected and analyzed for surface protein expression by flow cytometry. All experiments were repeated for three independent experiments, n=3. IFN = interferon gamma. Trame = trametinib. MFI are x 10^6. Statistical analysis were done using one-way ANOVA and Tukey post test comparison, where * indicates statistically significant changes, $p<0.05$. Events were gated for live singlets.

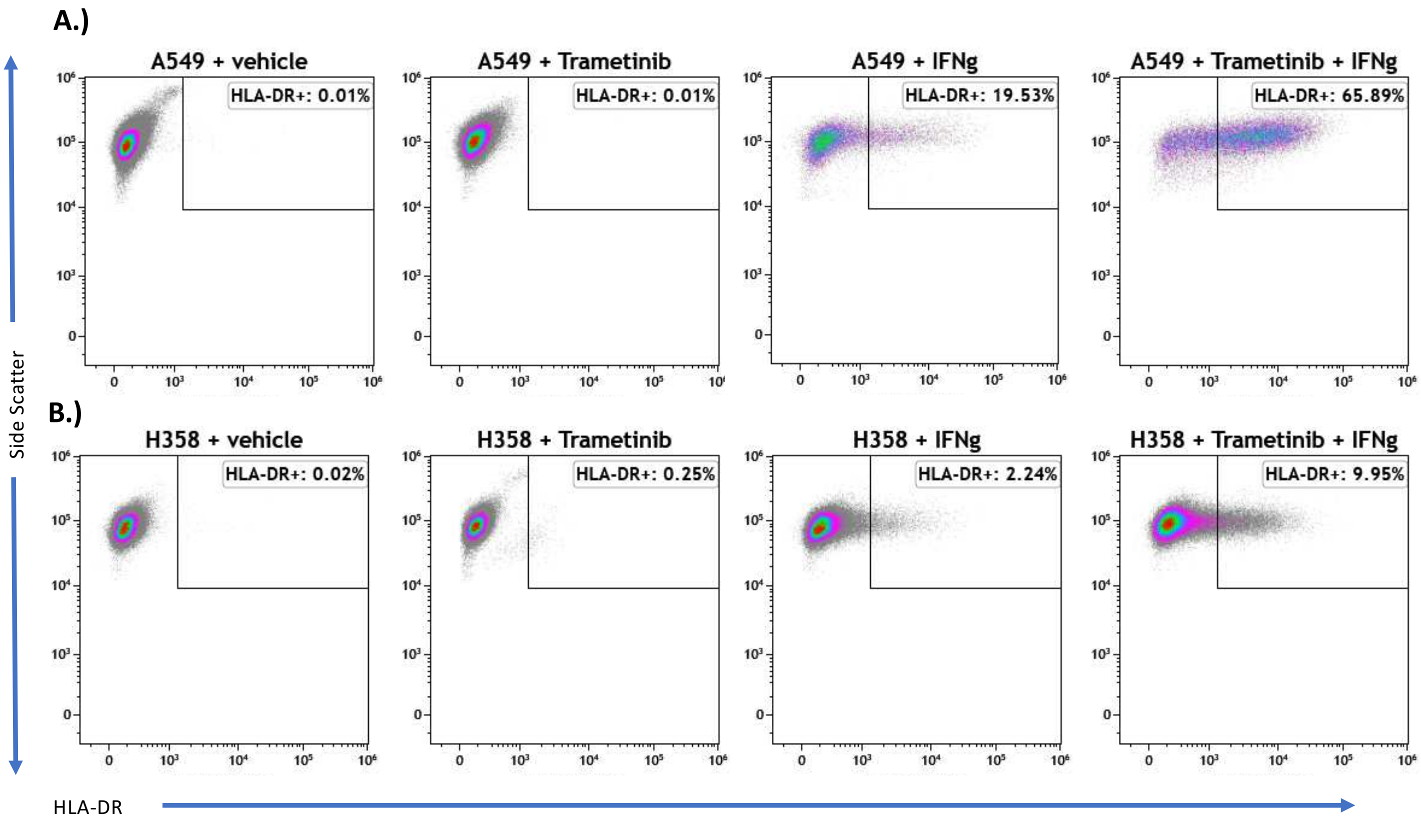
A.)
A549 + vehicle
HLA-DR+: 0.01%
A549 + Trametinib
HLA-DR+: 0.01%
A549 + IFNg
HLA-DR+: 19.53%
A549 + Trametinib + IFNg
HLA-DR+: 65.89%
B.)
H358 + vehicle
HLA-DR+: 0.02%
H358 + Trametinib
HLA-DR+: 0.25%
H358 + IFNg
HLA-DR+: 2.24%
H358 + Trametinib + IFNg
HLA-DR+: 9.95%
Side Scatter
HLA-DR

Supplemental Figure 8: Trametinib enhances the IFNγ-dependent induction of MHC II in some human NSCLC cell lines. The human NSCLC lines A549 (panel A) or H358 (panel B) were treated with 10nM of trametinib, 10 ng/mL of human recombinant IFNγ or a combination for 72 hours, after which, cells were analyzed for cell surface expression of HLA-DR by flow cytometry. Events were gated for live singlets.

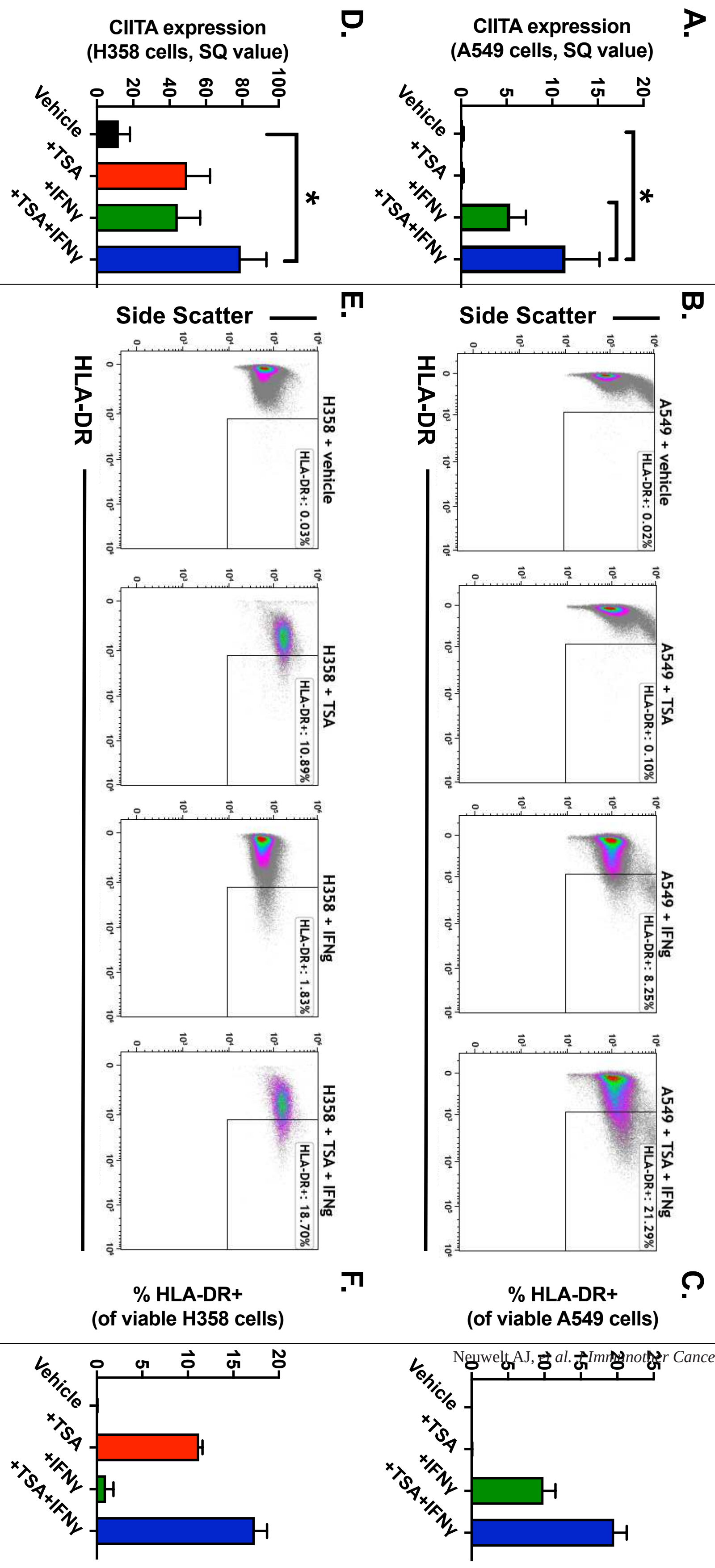
A.
CIITA expression
(A549 cells, SQ value)
0
5
10
15
20
Vehicle
+TSA
+IFNγ
+TSA+IFNγ
*
B.
Side Scatter
HLA-DR
A549 + vehicle
HLA-DR+: 0.02%
A549 + TSA
HLA-DR+: 0.10%
A549 + IFNg
HLA-DR+: 8.25%
A549 + TSA + IFNg
HLA-DR+: 21.29%
C.
% HLA-DR+
(of viable A549 cells)
0
5
10
15
20
25
Vehicle
+TSA
+IFNγ
+TSA+IFNγ
D.
CIITA expression
(H358 cells, SQ value)
0
20
40
60
80
100
Vehicle
+TSA
+IFNγ
+TSA+IFNγ
*
E.
Side Scatter
HLA-DR
H358 + vehicle
HLA-DR+: 0.03%
H358 + TSA
HLA-DR+: 10.89%
H358 + IFNg
HLA-DR+: 1.83%
H358 + TSA + IFNg
HLA-DR+: 18.70%
F.
% HLA-DR+
(of viable H358 cells)
0
5
10
15
20
Vehicle
+TSA
+IFNγ
+TSA+IFNγ

Supplemental Figure 9: Trichostatin A treatment enhances the IFNγ-dependent induction of MHC II in some human NSCLC cell lines. The human NSCLC lines A549 (panels A-C) or H358 (panel D-F) were treated with 100nM of TSA, 10ng/mL of human recombinant IFNγ. Cells were harvested at either 24 hours for qRT-PCR (panels A, D) or at 72 hours for flow cytometric analysis of cell surface expression of HLA-DR (panels B, C, E, F). Data depict mean +/- SEM from 3 independent experiments (A, D) or mean with range from 2 independent experiments (C, F). Cells were analyzed for cell surface expression of HLA-DR by flow cytometry. Events were gated for live singlets. Statistical analysis was done for panel A, D using one-way ANOVA, with statistically significant differences indicated by an asterisk, $p < 0.05$.

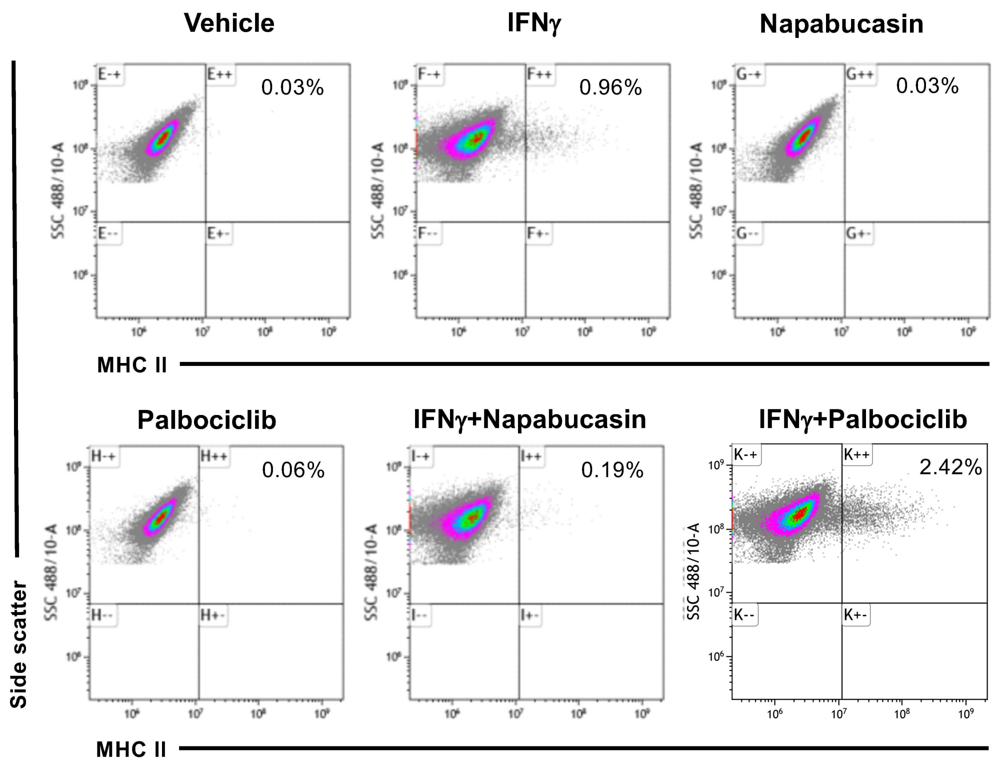
Vehicle
IFNγ
Napabucasin
0.03%
0.96%
0.03%
Palbociclib
IFNγ+Napabucasin
IFNγ+Palbociclib
0.06%
0.19%
2.42%
SSC 488/10-A
Side scatter
MHC II
MHC II

Supplemental Figure 10: Napabucasin and palbociclib have minimal effects on MHC II expression in LLC cells. LLC cells were treated for 48 hours with 1 μM napabucasin, 500 nM palbociclib, 100 ng/mL interferon gamma (IFNγ), or 1 μM napabucasin + IFNγ, or 500 nM palbociclib + IFNγ. Cells were then collected and analyzed by flow cytometry for cell surface expression of MHC II. Events were gated on single, viable cells, with the percentage of MHC II+ events in the right upper quadrant as indicated.
